# Dose-Related Effects of Different Tai Chi Styles Versus Traditional Community-Based Exercises on Cardiometabolic Health and Physical Function in Middle-Aged and Older Adults: Randomized Controlled Trial

**DOI:** 10.2196/80125

**Published:** 2026-04-23

**Authors:** Jiadong Qiu, Jian Wang, Xiongying Song, Wanyu Shu, Sung Min Kim

**Affiliations:** 1 Department of Sports Science Hanyang University Seoul Republic of Korea; 2 Department of Orthopedics Beijing Fengtai Hospital Beijing China; 3 Department of Active Aging Industry Hanyang University Seoul Republic of Korea; 4 BK21 FOUR Human-Tech Convergence Program Hanyang University Seoul Republic of Korea; 5 Center for Artificial Intelligence Muscle Hanyang University Seoul Republic of Korea

**Keywords:** Tai Chi, cardiometabolic health, functional rehabilitation, community-dwelling population, middle-aged and older adults

## Abstract

**Background:**

Age-related declines in metabolic, cardiovascular, and physical function contribute to reduced quality of life in older adults. Although structured exercise is central to healthy aging, the optimal modality remains unclear. Community-based exercise programs in China are heterogeneous, and their comparative effects on health outcomes and cardiovascular safety have not been systematically evaluated.

**Objective:**

We aimed to compare the effects of 4 common community-based exercise modalities on cardiometabolic health, physical function, and quality of life in middle-aged and older adults, and to assess their relative efficacy and safety across intervention exposure.

**Methods:**

This single-blind randomized controlled trial included 113 middle-aged and older adults (mean age 62.3, SD 4.25 years). Participants were assigned to one of the 5 groups: 12-form Chen-style Tai Chi (CTC12), 24-form Tai Chi, square dance, walking, or a control group. The 12-week intervention comprised 2 supervised sessions per week, each lasting 90 minutes. Pre- and postintervention assessments included blood pressure, lipid profiles, fasting glucose, interleukin-6 levels, Short Physical Performance Battery (SPPB) scores, activities of daily living, and the World Health Organization Quality of Life (WHOQOL) index.

**Results:**

The CTC12 and square dance groups showed significant improvements in several outcomes. In the CTC12 group, triglyceride and low-density lipoprotein cholesterol levels significantly decreased (*P*=.008 and *P*=.002, respectively), whereas SPPB and WHOQOL scores significantly improved (*P*=.02 and *P*=.002, respectively). In the square dance group, total cholesterol, triglyceride, and low-density lipoprotein cholesterol levels significantly decreased (*P*=.01, *P*=.002, and *P*=.002, respectively), whereas SPPB, activities of daily living, and WHOQOL scores significantly improved (*P*=.04, *P*=.04, and *P*=.002, respectively). The walking group showed a significant improvement only in WHOQOL scores (*P*=.02). No significant changes were observed in the control group for any outcome.

**Conclusions:**

CTC12 and square dance were associated with significant improvements in cardiometabolic, physical, and psychological health outcomes in middle-aged and older adults. CTC12 demonstrated broader multidimensional benefits, potentially reflecting the integration of physical, respiratory, and cognitive components. These modalities may represent safe and scalable strategies for promoting healthy aging in community settings.

**Trial Registration:**

Chinese Clinical Trial Registry ChiCTR2400092473; https://www.chictr.org.cn/showprojEN.html?proj=249330

## Introduction

The global population is aging at an unprecedented rate, with the number of individuals aged >65 years worldwide projected to increase from 703 million in 2019 to 1.5 billion by 2050 [1,2]. As life expectancy rises, older adults are increasingly susceptible to chronic conditions and their associated health burdens [3], a shift that has contributed to higher rates of chronic disease, poor health status, and functional decline among middle-aged and older populations [4,5]. Multimorbidity further compounds this burden, affecting approximately 46% of older adults worldwide [6-8], thereby underscoring the need to prioritize preventive strategies. Among these, exercise remains a key nonpharmacological intervention with well-established benefits for cardiometabolic health in aging populations [9,10].

Advancing age is associated with an increased risk of cardiovascular diseases, including hypertension and hyperlipidemia, as well as metabolic disorders, such as metabolic syndrome and diabetes mellitus (DM) [11,12]. Hypertension, affecting approximately 30% of the population and disproportionately prevalent in older adults, is a major contributor to global mortality [13-15]. Elevated systolic blood pressure (SBP) and diastolic blood pressure (DBP) are well-established predictors of cardiovascular disease and all-cause mortality [16], while pulse pressure (PP), reflecting the interplay between SBP and DBP, has gained recognition as an important indicator of vascular aging and mortality risk [17]. Dyslipidemia—characterized by elevated total cholesterol (TC) and low-density lipoprotein cholesterol (LDL-C), alongside reduced high-density lipoprotein cholesterol (HDL-C)—is likewise strongly associated with adverse outcomes in older populations [18]. Additionally, composite indices integrating glucose and lipid metabolism, including fasting plasma glucose (FPG) and the triglyceride/HDL-C ratio, have emerged as important predictors of cardiometabolic risk [19].

DM, a chronic metabolic noncommunicable disease characterized by persistent hyperglycemia, remains a significant contributor to global mortality [20-22], with diagnosis defined by FPG levels≥7.0 mmol/L (126 mg/dL) [23]. In 2015, approximately 415 million adults aged 20-79 years had diabetes, and this number is projected to increase by an additional 200 million by 2040 [20], placing a growing strain on health care systems worldwide, particularly among older populations [12]. In parallel, age-related chronic low-grade inflammation, commonly termed “inflammaging” [24], is characterized by maintained elevations in proinflammatory markers, such as interleukin-6 (IL-6), and is closely linked to cardiovascular disease, diabetes, and metabolic syndrome [25,26]. Accordingly, IL-6 was used in this study as a biomarker to capture the systemic physiological effects of exercise. Beyond physical health, older adults face an increased risk of mental health challenges [27,28], necessitating a comprehensive evaluation of both functional and psychological domains. Thus, we used validated instruments, including the Short Physical Performance Battery (SPPB), activities of daily living (ADLs), and the World Health Organization Quality of Life (WHOQOL) index to assess multidimensional outcomes [29-31]. Midlife represents a critical period during which risk factors for functional decline emerge, while also offering substantial potential for recovery [32]. Thus, we focused on middle-aged and older adults to examine the role of exercise in disease prevention and functional rehabilitation.

Tai Chi, originating from traditional Chinese medicine and guided by movement-based theoretical frameworks, integrates principles, such as yin-yang philosophy and meridian theory [33]. It has evolved into a comprehensive practice promoting both physical and psychological well-being. Empirical evidence indicates that Tai Chi confers benefits across a range of conditions, including hypertension, hyperlipidemia, DM, metabolic syndrome, inflammation, impaired physical function, limitations in ADLs, and psychological disorders [34-36]. However, most interventions have relied on the simplified 24-form Tai Chi (TC24), derived from Yang-style Tai Chi [37], which emphasizes slow, low-intensity movements and simplified structures that facilitate safety and widespread dissemination.

Despite these advantages, its low intensity and repetitive movements may limit energy expenditure and neuromuscular engagement, potentially constraining its effectiveness in improving cardiometabolic health and higher-level physical function [38,39].

These limitations highlight the necessity for more dynamic and integrative exercise modalities [40]. Chen-style Tai Chi incorporates both slow, controlled movements and rapid, forceful actions, including stomping and explosive movements, thereby enhancing neuromuscular activation and energy metabolism while maintaining safety and fluidity of motion [41,42]. The 12-form Chen-style Tai Chi (CTC12) preserves the core elements of traditional Chen-style practice while integrating 12-meridian regulation theory from traditional Chinese exercise, resulting in a rhythmically diverse and functionally comprehensive intervention. CTC12 is hypothesized to provide greater benefits than TC24 in improving neuromuscular function, metabolic regulation, and multidimensional health outcomes in middle-aged and older adults [43].

In parallel, square dance represents a widely adopted form of aerobic exercise among middle-aged and older populations, offering both physical and psychosocial benefits in community settings [44]. Similarly, walking, another accessible and low-risk group-based activity, has gained popularity. Both modalities are associated with lower cardiovascular demands and risk of injury, and have been proven efficient in improving blood pressure, lipid metabolism, glucose regulation, and psychological well-being, including reductions in symptoms of depression and anxiety [33,45].

Despite evidence supporting the benefits of Tai Chi and aerobic exercise for cardiovascular and metabolic health among older adults, several gaps remain. First, most studies have focused on TC24, with limited investigation of more dynamic forms, such as Chen-style Tai Chi, and variability in intervention characteristics—including duration, intensity, and form—has contributed to heterogeneity in reported outcomes, underscoring the need for more standardized approaches [46]. Second, previous research has often examined single health outcomes, with limited evaluation of multidimensional physiological effects, particularly inflammatory markers [47]. Third, comparative studies of community-based aerobic activities, including Tai Chi, square dance, and walking, remain limited, and direct comparisons with structured mind-body interventions are scarce. Addressing these gaps requires comprehensive, well-designed randomized controlled trials [48,49].

Accordingly, this study was conducted to systematically evaluate the effects of CTC12, TC24, square dance, and walking on cardiometabolic, functional, and psychological outcomes in community-dwelling middle-aged and older adults, while assessing the safety and dose-related efficacy of each modality. Moreover, the study sought to provide evidence to inform the development of safe, effective, and sustainable individualized exercise strategies for aging populations.

## Methods

### Participants

In this study, we enrolled community-dwelling middle-aged and older adults who were capable of participating in physical exercise. Participants were recruited from local community centers and health clinics. Of 155 individuals screened, 120 were enrolled (mean age 63.0, SD 4.5 years; 97/120, 80.8% female). Eligibility criteria included age 50-70 years, absence of clinically diagnosed cardiovascular, musculoskeletal, metabolic, or cognitive disorders, and no regular participation in structured exercise within the preceding 6 months (defined as <1 session/week). Exclusion criteria comprised current use of medications affecting blood pressure, lipid metabolism, or glucose regulation (eg, antihypertensive agents, statins, or antidiabetic drugs), as well as current smoking or smoking cessation within the past 6 months.

### Study Design

This study was a single-blind randomized controlled trial conducted among community-dwelling middle-aged and older adults. Following baseline assessments, participants were randomly assigned in a 1:1:1:1:1 ratio to one of the 5 groups—CTC12, TC24, square dance, walking, or a control group. Randomization was performed using a computer-generated sequence prepared by an independent researcher, with allocation concealed in sequentially numbered, opaque, sealed envelopes, which were opened after baseline assessments. Outcome assessors and laboratory personnel were blinded to group allocation, and participants were informed only of their assigned intervention. Each participant received a unique identification code, and all assessments were conducted according to standardized procedures by trained evaluators. No sex-based restrictions were applied, and all interventions and assessments were implemented uniformly across sexes. Before the intervention, participants completed a 1-week familiarization period to facilitate adaptation, enhance adherence, and ensure correct execution of the assigned exercises.

### Ethical Considerations

The study protocol was approved by the Ethics Committee of Beijing Fengtai Hospital (approval P2024022) and complied with the Declaration of Helsinki. All participants provided written informed consent after receiving detailed information regarding study objectives, procedures, potential risks, and participant rights. Assessments were conducted by licensed medical professionals from Beijing Fengtai Hospital and Nankang District Hospital of Traditional Chinese Medicine (Ganzhou, Jiangxi Province). Adverse events were evaluated and managed on-site. Personal data were anonymized and coded to ensure confidentiality and were used exclusively for research purposes.

After completing the study, participants were provided with full electronic instructional videos for CTC12, TC24, and square dancing.

### Intervention Protocol

Participants in each intervention group attended supervised sessions twice weekly for 90 minutes over a 12-week period, conducted from 8 to 9:30 AM. The interventions were defined as follows: (1) CTC12, which emphasized explosive techniques (fa jin), jumps, and complex transitions to enhance neuromuscular coordination and cardiopulmonary function; (2) TC24, a standardized and simplified form of Yang-style Tai Chi, focusing on breathing rhythm, flexibility, and balance [37]; (3) square dance, a moderate-to-high intensity aerobic dance performed at 60%-75% of maximum heart rate (HR) [50]; (4) walking, performed as brisk walking at 3.5-4.0 km/h on flat terrain, targeting 50%-60% of maximum HR, and monitored using HR devices [50,51]; and (5) control, continued daily activities with no additional structured exercise.

All sessions were conducted outdoors under temperatures ranging from 24 °C to 28 °C. Although humidity and air circulation were not controlled, participant safety and comfort were ensured throughout. Each session began with a standardized 10-minute warm-up consisting of light aerobic activity and joint mobility exercises. Tai Chi interventions (CTC12 and TC24) were led by Shaoqing Zeng, with more than 10 years of teaching experience and recognized competitive achievements at the city level. Square dance and walking interventions were designed and supervised by WS, with a doctoral degree in Sports Science and extensive experience in physical education and exercise interventions, who had participated in the design and implementation of several community-based exercise programs (Multimedia Appendix 1).

### Outcome Measures

#### Primary Outcomes

Fasting venous blood samples were collected between 7 AM and 9 AM before and after the 12-week intervention, following ≥10 hours of fasting, and were processed within 2 hours. Blood pressure was measured twice in a seated position using an automated sphygmomanometer (Omron HEM-7320 [Omron Healthcare]), and PP was calculated as SBP−DBP [52]. Lipid profiles, including TC, triglycerides, HDL-C, and LDL-C, were measured using enzymatic colorimetric assays (Cobas c 501 [Roche]) [52,53]. FPG was measured using the glucose oxidase method (LabX) [54]. Serum IL-6 concentrations were quantified using a high-sensitivity ELISA kit (Human IL-6 Quantikine HS ELISA [R&D Systems]; catalog HS600C) [55], with a detection limit of 0.09 pg/mL. According to the manufacturer’s specifications, the intra-assay coefficient of variation ranged from 3.6% to 4.7% and interassay coefficient of variation from 3.9% to 10.8% across 3 concentration levels. Serum samples were aliquoted and stored at −80 °C until analysis [56]. Samples were assayed in duplicate, and the mean of the 2 measurements was used for analysis. The assay detection limit was 0.09 pg/mL. Laboratory technicians were blinded to group allocation, and all samples were analyzed in a single batch to minimize variability.

Basal metabolic rate (BMR) was estimated using bioelectrical impedance analysis (InBody H30NWi [InBody Co]) [57]. HR data, including resting HR and average exercise HR, were recorded using photoplethysmography-based monitors (Polar H10 [Polar Electro]) [58]. The change in heart rate (ΔHR) was defined as the difference between average exercise HR and resting HR during each 90-minute session. Energy expenditure (kcal), including calories at rest and additional calories expended during exercise sessions, was estimated from continuous HR monitoring (Polar H10) [59].

Physical function was assessed using the SPPB (score range 0-12), which includes static balance, 4-meter gait speed, and the 5-time chair stand test [60]. Functional independence was evaluated using ADLs, measured as the ability to perform basic daily activities [61]. WHOQOL was assessed using total scores across 6 domains from the WHOQOL-100 instrument [62].

#### Secondary Outcomes

Secondary outcomes included the six WHOQOL domain scores: (1) Q1: physical health, (2) Q2: psychological health, (3) Q3: social relationships, (4) Q4: environment, (5) Q5: self-perceived health, and (6) Q6: self-rated overall quality of life [62,63]. All evaluations were conducted by trained personnel blinded to group assignment.

#### Sample Size Estimation

An a priori power analysis was performed using G*Power (version 3.1.9.7) for a mixed repeated-measures ANOVA (within-between interaction; group×time interaction). The SPPB was selected as the primary outcome, reflecting the central role of physical function in this study. The expected effect size was set at η^2^=0.065 based on previous literature [64], corresponding to Cohen *f*=√(η^2^/(1−η^2^))=0.264. Assuming α=.05, power (1−β)=0.95, 5 groups, 2 measurements (pre- and postintervention), correlation among repeated measures of 0.50, and ε=1, the required sample size was 75 participants (15 per group). To account for an anticipated attrition rate of 20%, a minimum of 94 participants (required final sample size=75, expected retention rate=0.80) were targeted (Multimedia Appendix 2).

### Statistical Analysis

All statistical analyses were performed using R (version 4.2.1; R Core Team). Continuous variables are presented as mean (SD). Within-group comparisons were assessed using paired *t* tests, and between-group comparisons were conducted using 1-way ANOVA with Tukey post hoc tests. Normality and homogeneity of variance were evaluated using the Shapiro-Wilk and Levene tests, respectively. To address potential inflation of type I error due to multiple comparisons across outcomes (eg, blood pressure, fasting glucose, lipids, IL-6, SPPB, and WHOQOL subdomains), the false discovery rate correction was applied. All tests were 2-tailed, with statistical significance defined as *P*<.05. Adjusted *P* values, effect sizes, and 95% CIs were reported to complement dichotomous significance testing.

## Results

### Participant Flow and Baseline Characteristics

**Figure 1:**
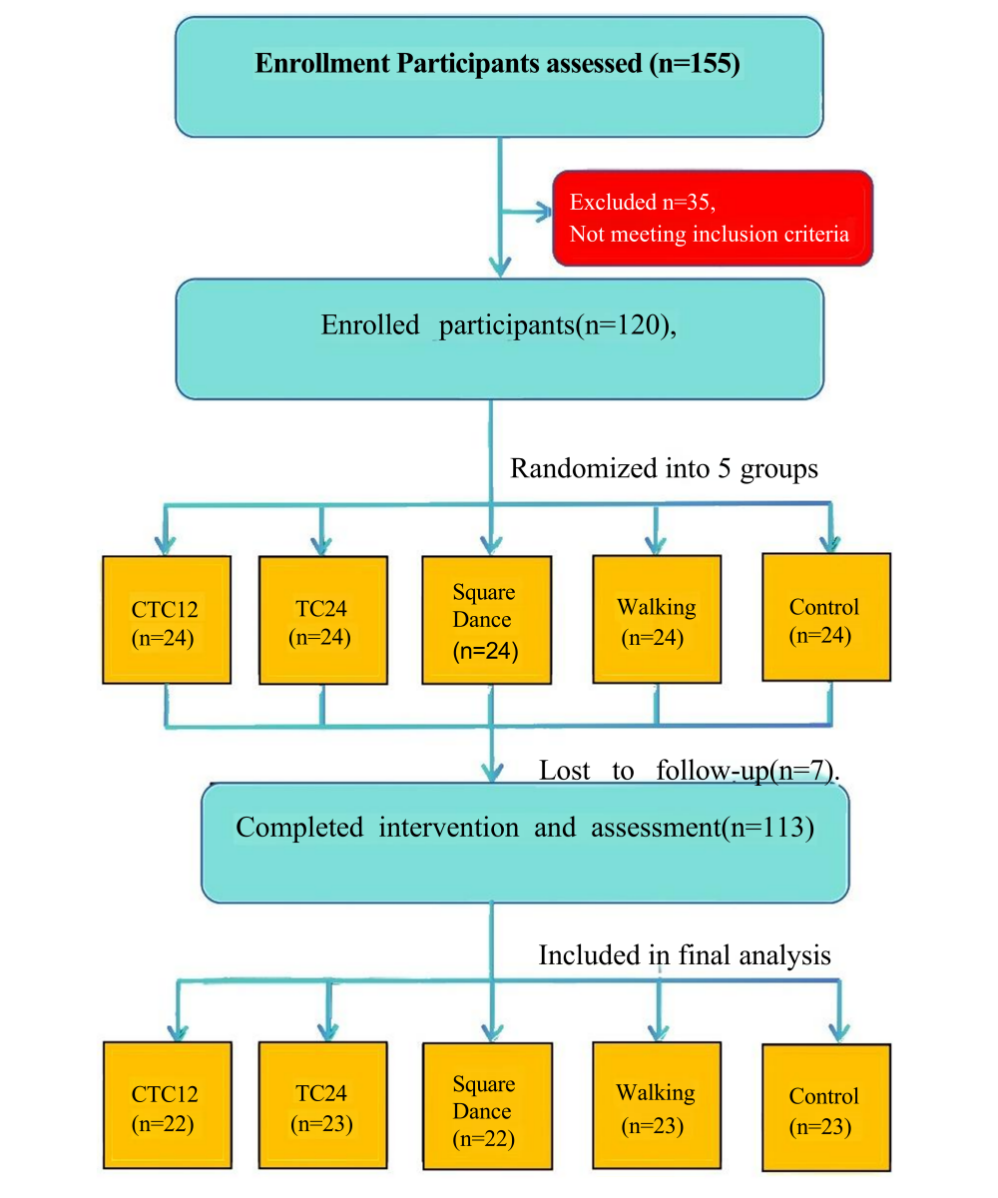
CONSORT (Consolidated Standards of Reporting Trials) flow diagram of participant recruitment, group allocation, intervention, and analysis. Control: control group; CTC12: Chen-style Tai Chi 12 forms group; Square Dance: square dance group; TC24: Simplified Tai Chi 24 forms group; Walking: walking exercise group. All analyzed participants attended ≥90% of sessions and met protocol compliance.

As illustrated in the CONSORT (Consolidated Standards of Reporting Trials) diagram, 155 individuals were screened, of whom 120 (77.4%) eligible community-dwelling middle-aged and older adults were enrolled. During the 12-week intervention, 7 participants withdrew due to personal reasons or insufficient attendance (<90%) and were subsequently classified as dropouts. Ultimately, 113 participants completed the intervention and all assessments. The final group sizes were as follows: (1) CTC12: n=22, (2) TC24: n=23, (3) square dance: n=22, (4) walking: n=23, and (5) control: n=23 (Figure 1, Table 1).

**Table 1:** Baseline characteristics of participants across intervention groups.

Variable		CTC12^a^ group (n=22), mean (SD)	TC24^b^ group (n=23), mean (SD)	Square dance group (n=22), mean (SD)	Walking group (n=23), mean (SD)	Control group (n=23), mean (SD)	*P* value^c^
Age (y)		62.45 (5.26)	61.65 (2.99)	62.64 (4.11)	61.61 (4.29)	63.17 (4.48)	.69
**Sex, n**							
	Male	5	5	4	5	4	.99
	Female	17	18	18	18	19	.99
Height (cm)		159.14 (6.07)	158.22 (5.00)	158.32 (5.11)	158.83 (5.04)	160.13 (4.86)	.74
Weight (kg)		60.42 (8.39)	59.09 (6.96)	59.11 (6.38)	59.38 (6.73)	61.47 (6.82)	.75
BMI (kg/m²)		23.87 (3.20)	23.59 (2.35)	23.62 (2.80)	23.51 (2.00)	23.98 (2.54)	.97
DBP^d^ (mm Hg)		80.59 (6.81)	81.87 (5.52)	81.09 (6.24)	80.43 (7.04)	80.87 (6.06)	.95
SBP^e^ (mm Hg)		126.18 (7.69)	126.04 (7.74)	126.05 (6.42)	126.43 (9.85)	125.70 (7.78)	.99
PP^f^ (mm Hg)		45.59 (9.05)	44.17 (8.89)	44.96 (7.51)	46.00 (7.67)	44.83 (7.77)	.95
TC^g^ (mmol/L)		5.40 (0.97)	5.44 (1.05)	5.43 (1.06)	5.39 (0.82)	5.51 (1.01)	.99
TG^h^ (mmol/L)		1.62 (1.03)	1.70 (0.82)	1.62 (0.52)	1.66 (0.66)	1.70 (0.61)	.99
HDL-C^i^ (mmol/L)		1.46 (0.30)	1.37 (0.35)	1.30 (0.30)	1.34 (0.33)	1.30 (0.18)	.35
LDL-C^j^ (mmol/L)		2.67 (0.57)	2.71 (0.64)	2.60 (0.54)	2.62 (0.59)	2.74 (0.73)	.94
Fasting glucose (mmol/L)		5.28 (0.67)	5.32 (0.85)	5.26 (0.81)	5.30 (0.81)	5.38 (0.81)	.99
IL-6^k^ (pg/ml)		2.91 (1.88)	2.90 (1.75)	2.99 (1.84)	2.93 (2.50)	2.99 (1.50)	.52
BMR^l^ (kcal)		1273.32 (108.56)	1322.24 (114.76)	1271.36 (60.90)	1261.61 (103.29)	1264.78 (99.30)	.23
SPPB^m^ (scores)		9.59 (0.67)	9.52 (0.99)	9.50 (1.10)	9.57 (0.59)	9.35 (0.83)	.89
ADL^n^ (scores)		98.64 (2.28)	98.70 (2.24)	98.18 (2.46)	98.70 (2.24)	98.91 (2.11)	.87
WHOQOL^o^ total (scores)		102.36 (7.49)	102.26 (5.77)	102.14 (6.22)	101.87 (5.41)	102.13 (5.27)	.10
Resting heart rate (bpm)		67.77 (3.64)	67.83 (3.88)	67.59 (3.98)	67.70 (4.76)	67.22 (3.58)	.99
Calories at rest (kcal)		84.38 (5.82)	83.20 (6.67)	83.68 (4.57)	83.01 (6.54)	84.03 (6.74)	.94

^a^CTC12: 12-form Chen-style Tai Chi.

^b^TC24: 24-form simplified Tai Chi.

^c^No significant differences were observed between groups at baseline (*P*>.05 for all comparisons).

^d^DPB: diastolic blood pressure.

^e^SPB: systolic blood pressure.

^f^PP: pulse pressure.

^g^TC: total cholesterol.

^h^TG: triglycerides.

^i^HDL-C: high-density lipoprotein cholesterol.

^i^LDL-C: low-density lipoprotein cholesterol.

^k^IL-6: interleukin-6.

^l^BMR: basal metabolic rate.

^m^SPPB: Short Physical Performance Battery.

^n^ADL: activity of daily living.

^o^WHOQOL: World Health Organization Quality of Life questionnaire.

### Primary Outcomes

#### Within-Group Comparisons

No significant changes were observed in DBP, SBP, and PP across all groups (all *P*>.05). Regarding lipid profiles, TC significantly decreased only in the square dance group (*P*=.01). Triglyceride and LDL-C significantly decreased in both the CTC12 (*P*=.008 and *P*=.002, respectively) and square dance (*P*=.002 for both) groups. HDL-C remained unchanged in all groups (all *P*>.05). In terms of fasting glucose and IL-6, no significant changes were noted in any group (all *P*>.05). For physical function and quality of life, SPPB scores significantly improved in the CTC12 (*P*=.02) and square dance (*P*=.04) groups, whereas ADL scores significantly improved only in the square dance group (*P*=.04). WHOQOL scores significantly improved in the CTC12 (*P*=.002), square dance (*P*=.002), and walking (*P*=.02) groups (Table 2).

**Table 2:** Within-group comparisons of key health indicators before and after intervention (paired *t* tests).

Variable and group			Preintervention, mean (SD)		Postintervention, mean (SD)		*t* test (*df*)		Change^a^, marginal mean (95% CI)		*P* value		Adjusted *P* value
**DBP^b^ (mm Hg)**													
	CTC12^c^	80.59 (6.81)		80.36 (6.92)		–0.6918 (21)		–0.23 (–0.91 to 0.46)		.50		.62	
	TC24^d^	81.87 (5.52)		81.65 (5.73)		–0.7740 (22)		1.26 (–0.80 to 0.37)		.45		.62	
	Square dance	81.09 (6.24)		81.36 (6.18)		1.2396 (21)		0.27 (–0.18 to 0.73)		.23		.48	
	Walking	80.43 (7.04)		79.61 (6.85)		–2.7018 (22)		–0.82 (–1.46 to –0.19)		.01		.13	
	Control	80.87 (6.06)		80.83 (5.67)		–0.2041 (22)		–0.04 (–0.49 to 0.40)		.84		.84	
**SBP^e^ (mm Hg)**													
	CTC12	126.18 (7.69)		125.45 (7.25)		–2.3470 (21)		–0.73 (–1.37 to –0.08)		.03		.14	
	TC24	126.04 (7.74)		125.57 (7.50)		–0.7359 (22)		–0.47 (–1.83 to 0.87)		.47		.62	
	Square dance	126.05 (6.42)		125.59 (6.53)		–1.2076 (21)		–0.46 (–1.24 to 0.33)		.24		.48	
	Walking	126.43 (9.85)		126.30 (9.78)		–0.3708 (22)		–0.13 (–0.86 to 0.40)		.71		.79	
	Control	125.70 (7.78)		125.96 (7.57)		1.3667 (22)		0.26 (–0.13 to 0.66)		.19		.48	
**PP^f^ (mm Hg)**													
	CTC12	45.59 (9.05)		45.09 (9.01)		–1.7100 (21)		–0.50 (–1.12 to 0.12)		.10		.15	
	TC24	44.17 (8.89)		43.91 (8.54)		–0.3800 (22)		–0.26 (–1.68 to 1.16)		.70		.75	
	Square dance	44.96 (7.51)		44.23 (7.78)		–2.5238 (21)		–0.73 (–1.33 to –0.13)		.02		.07	
	Walking	46.00 (7.67)		46.70 (7.61)		1.8085 (22)		0.70 (–0.10 to 1.49)		.08		.13	
	Control	44.83 (7.77)		45.09 (7.65)		2.0205 (22)		0.26 (–0.01 to 0.53)		.06		.11	
**TC^g^ (mmol/L)**													
	CTC12	5.40 (0.97)		5.03 (0.63)		–2.7694 (21)		–0.37 (–0.65 to –0.09)		.01		.05	
	TC24	5.44 (1.05)		5.28 (0.99)		–1.2498 (22)		–0.16 (–0.42 to 0.10)		.23		.30	
	Square dance	5.43 (1.06)		4.79 (1.16)		–3.5620 (21)		–0.64 (–1.00 to –0.26)		.002		.01	
	Walking	5.39 (0.82)		5.24 (0.71)		–0.9954 (22)		–0.15 (–0.46 to 0.16)		.33		.39	
	Control	5.51 (1.01)		5.72 (0.84)		1.9356 (22)		0.21 (–0.01 to 0.42)		.07		.12	
**TG^h^ (mmol/L)**													
	CTC12	1.62 (1.03)		1.18 (0.60)		–3.9299 (21)		–0.44 (–0.68 to –0.21)		.001		.008	
	TC24	1.69 (0.82)		1.49 (0.77)		–2.7039 (22)		–0.20 (–0.37 to –0.05)		.01		.05	
	Square dance	1.62 (0.52)		1.27 (0.34)		–4.6725 (21)		–0.35 (–0.50 to –0.19)		<.001		.002	
	Walking	1.66 (0.66)		1.48 (0.67)		–2.4388 (22)		–0.18 (–0.34 to –0.03)		.023		.067	
	Control	1.70 (0.61)		1.93 (0.68)		1.8613 (22)		0.23 (–0.03 to 0.47)		.09		.13	
**HDL-C^i^ (mmol/L)**													
	CTC12	1.47 (0.30)		1.53 (0.30)		2.1168 (21)		0.06 (0.00 to 0.13)		.05		.11	
	TC24	1.37 (0.35)		1.38 (0.37)		0.2423 (22)		0.01 (–0.076 to 0.10)		.81		.81	
	Square dance	1.30 (0.30)		1.37 (0.37)		2.0256 (21)		0.07 (–0.00 to 0.13)		.06		.11	
	Walking	1.34 (0.33)		1.37 (0.26)		1.1281 (22)		0.03 (–0.03 to 0.09)		.27		.34	
	Control	1.30 (0.18)		1.27 (0.19)		–0.9172 (22)		–0.03 (–0.12 to 0.05)		.37		.41	
**LDL-C^j^ (mmol/L)**													
	CTC12	2.67 (0.57)		2.23 (0.70)		–4.6953 (21)		–0.44 (–0.64 to –0.25)		<.001		.002	
	TC24	2.71 (0.64)		2.57 (0.54)		–2.3375 (22)		–0.14 (–0.26 to –0.02)		.03		.07	
	Square dance	2.60 (0.54)		2.19 (0.48)		–4.5876 (21)		–0.41 (–0.60 to –0.23)		<.001		.002	
	Walking	2.62 (0.59)		2.50 (0.56)		–2.2419 (22)		–0.12 (–0.25 to –0.01)		.04		.08	
	Control	2.74 (0.73)		2.91 (0.77)		1.7128 (22)		0.17 (–0.035 to 0.37)		.10		.15	
**Fasting glucose (mmol/L)**													
	CTC12	5.35 (0.80)		5.06 (0.56)		–2.4815 (21)		–0.29 (–0.53 to –0.05)		.02		.07	
	TC24	5.32 (0.85)		5.28 (0.77)		–0.4939 (22)		–0.04 (–0.23 to 0.14)		.63		.67	
	Square dance	5.26 (0.81)		4.89 (0.82)		–2.1352 (21)		–0.37 (–0.72 to –0.01)		.05		.09	
	Walking	5.30 (0.81)		5.26 (0.59)		–0.3960 (22)		–0.04 (–0.24 to 0.16)		.70		.72	
	Control	5.38 (0.81)		5.50 (0.75)		1.4302 (22)		0.12 (–0.05 to 0.29)		.17		.21	
**IL-6^k^ (pg/ml)**													
	CTC12	2.91 (1.88)		2.58 (1.46)		–2.3568 (21)		–0.33 (–0.62 to –0.04)		.03		.07	
	TC24	2.90 (1.75)		2.83 (1.69)		–0.5833 (22)		–0.07 (–0.34 to 0.19)		.57		.63	
	Square dance	2.99 (1.84)		2.88 (1.71)		–1.8769 (21)		–0.11 (–0.20 to 0.01)		.08		.14	
	Walking	2.93 (2.50)		2.87 (2.48)		–1.3773 (22)		–0.06 (–0.17 to 0.03)		.18		.22	
	Control	2.99 (1.50)		3.09 (1.42)		1.7851 (22)		0.10 (–0.02 to 0.22)		.09		.14	
**SPPB^l^ (scores)**													
	CTC12	9.59 (0.67)		10.00 (0.82)		3.2504 (21)		0.41 (0.15 to 0.67)		.004		.02	
	TC24	9.52 (0.99)		9.65 (0.88)		1.8166 (22)		0.13 (–0.02 to 0.28)		.08		.14	
	Square dance	9.50 (1.10)		10.00 (0.93)		2.9250 (21)		0.50 (0.14 to 0.86)		.008		.04	
	Walking	9.57 (0.59)		9.61 (0.84)		0.2955 (22)		0.04 (–0.26 to 0.35)		.77		.77	
	Control	9.35 (0.83)		9.22 (0.74)		–1.3667 (22)		–0.13 (–0.33 to 0.07)		.19		.22	
**ADL^m^ (scores)**													
	CTC12	98.64 (2.28)		99.55 (1.47)		2.1602 (21)		0.91 (0.03 to 1.78)		.04		.09	
	TC24	98.26 (2.43)		98.48 (2.35)		1.0000 (22)		0.22 (–0.23 to 0.67)		.33		.38	
	Square dance	98.18 (2.46)		99.55 (1.47)		2.8062 (21)		1.37 (0.35 to 2.37)		.01		.04	
	Walking	98.70 (2.24)		99.35 (1.72)		1.8166 (22)		0.65 (–0.09 to 1.40)		.08		.14	
	Control	98.91 (2.11)		98.48 (2.35)		–1.4475 (22)		–0.43 (–1.06 to 0.19)		.16		.22	
**WHOQOL^n^ total (scores)**													
	CTC12	102.36 (7.49)		105.23 (5.76)		4.4585 (21)		2.87 (1.53 to 4.20)		<.001		.002	
	TC24	102.26 (5.77)		104.65 (4.24)		2.3246 (22)		2.39 (0.26 to 4.52)		.03		.07	
	Square dance	102.14 (6.22)		105.18 (5.33)		4.6643 (21)		3.04 (1.69 to 4.40)		<.001		.002	
	Walking	101.87 (5.41)		103.48 (5.24)		3.2565 (22)		1.61 (0.58 to 2.63)		.004		.02	
	Control	102.13 (5.27)		101.43 (5.21)		–1.6599 (22)		–0.70 (–1.56 to 0.17)		.11		.16	

^a^Change indicates the mean difference between pre- and postintervention values.

^b^DPB: diastolic blood pressure.

^c^CTC12: 12-form Chen-style Tai Chi.

^d^TC24: 24-form simplified Tai Chi.

^e^SPB: systolic blood pressure.

^f^PP: pulse pressure.

^g^TC: total cholesterol.

^h^TG: triglycerides.

^i^HDL-C: high-density lipoprotein cholesterol.

^i^LDL-C: low-density lipoprotein cholesterol.

^k^IL-6: interleukin-6.

^l^SPPB: Short Physical Performance Battery.

^m^ADL: activity of daily living.

^n^WHOQOL: World Health Organization Quality of Life questionnaire.

#### Between-Group Comparisons

Postintervention differences were assessed using 1-way ANOVA with Tukey post hoc tests (Table 3; Figure S1 in Multimedia Appendix 3).

**Table 3:** Between-group comparisons of postintervention outcomes among 5 intervention arms (1-way ANOVA).

Variable	CTC12^a^ (n=22), mean (SD)	TC24^b^ (n=23), mean (SD)	Square dance (n=22), mean (SD)	Walking (n=23), mean (SD)	Control (n=23), mean (SD)	*F* test^c^ (*df*)	η^2d^	*P* value^e^	Post hoc^f^
DBP^g^ (mm Hg)	80.36 (6.92)	81.65 (5.73)	81.36 (6.18)	79.61 (6.85)	80.83 (5.67)	0.38 (4,108)	0.014	.82	Ns^h^
SBP^i^ (mm Hg)	125.45 (7.25)	125.57 (7.50)	125.59 (6.53)	126.30 (9.78)	125.91 (7.75)	0.04 (4,108)	0.002	.99	Ns
PP^j^ (mm Hg)	45.09 (9.01)	43.91 (8.54)	44.23 (7.78)	46.70 (7.61)	45.09 (7.65)	0.40 (4,108)	0.015	.81	Ns
TC^k^ (mmol/L)	5.03 (0.63)	5.28 (0.99)	4.79 (1.16)	5.24 (0.71)	5.79 (0.90)	3.82 (4,108)	0.124	.006	Control>CTC12, square dance
TG^l^ (mmol/L)	1.18 (0.60)	1.49 (0.77)	1.27 (0.34)	1.48 (0.67)	1.93 (0.68)	4.75 (4,108)	0.149	.001	Control>CTC12, square dance
HDL-C^m^ (mmol/L)	1.53 (0.30)	1.38 (0.37)	1.37 (0.37)	1.37 (0.27)	1.27 (0.19)	2.14 (4,108)	0.073	.08	Control<CTC12; others ns
LDL-C^n^ (mmol/L)	2.23 (0.70)	2.57 (0.54)	2.18 (0.47)	2.50 (0.56)	2.91 (0.77)	4.98 (4,108)	0.156	.001	Control>CTC12, square dance
Fasting glucose (mmol/L)	4.90 (0.58)	5.28 (0.77)	4.89 (0.82)	5.26 (0.59)	5.50 (0.75)	3.07 (4,108)	0.102	.02	Control>CTC12, square dance
IL-6^o^ (pg/ml)	2.58 (1.46)	2.83 (1.69)	2.88 (1.71)	2.87 (2.48)	3.09 (1.42)	0.23 (4,108)	0.009	.92	Ns
SPPB^p^ (scores)	10.00 (0.82)	9.61 (0.94)	10.00 (0.93)	9.61 (0.84)	9.22 (0.74)	3.31 (4,108)	0.109	.01	CTC12, square dance>Control
ADL^q^ (scores)	99.55 (1.47)	98.48 (2.35)	99.55 (1.47)	99.35 (1.72)	98.48 (2.35)	1.88 (4,108)	0.065	.12	Ns
WHOQOL^r^ total (scores)	105.23 (5.76)	104.65 (4.24)	105.18 (5.33)	103.48 (5.24)	100.91 (5.09)	2.80 (4,108)	0.094	.03	CTC12, square dance>control
BMR^s^ (kcal)	1391.50 (70.78)	1314.96 (91.49)	1396.55 (74.45)	1296.74 (102.82)	1257.22 (92.10)	10.90 (4,108)	0.2877	<.001	CTC12, square dance>control, TC24, walking
ΔHR^t^ (bpm)	29.50 (4.61)	22.48 (6.67)	46.68 (7.48)	19.48 (2.19)	0.00 (0.00)	105.35 (4,108)	0.7861	<.001	Square dance>CTC12> TC24 > Walking
1.5 hours additional calories burned (kcal)	363.75 (18.54)	207.80 (15.76)	386.65 (37.29)	166.36 (12.60)	0.00 (0.00)	518.87 (4,108)	0.948	<.001	Square dance>CTC12>TC24, Walking

^a^CTC12: 12-form Chen-style Tai Chi.

^b^TC24: 24-form simplified Tai Chi.

^c^*F* test: analysis of variance test statistic.

^d^η^2^: representing effect size.

^e^*P* values represent the significance of overall group differences.

^f^Pairwise comparisons were conducted using Tukey’s post hoc tests where appropriate.

^g^DPB: diastolic blood pressure.

^h^Ns: not significant.

^i^SPB: systolic blood pressure.

^j^PP: pulse pressure.

^k^TC: total cholesterol.

^l^TG: triglycerides.

^m^HDL-C: high-density lipoprotein cholesterol.

^n^LDL-C: low-density lipoprotein cholesterol.

^o^IL-6: interleukin-6.

^p^SPPB: Short Physical Performance Battery.

^q^ADL: activity of daily living.

^r^WHOQOL: World Health Organization Quality of Life questionnaire.

^s^BMR: basal metabolic rate.

^t^ΔHR: heart rate increase.

No significant between-group differences were observed for SBP (*P*=.99), DBP (*P*=.82), or PP (*P*=.81).

TC, triglyceride, and LDL-C were significantly lower in the CTC12 (mean 5.03, SD 0.63 mmol/L; mean 1.18, SD 0.60 mmol/L; and mean 2.23, SD 0.70 mmol/L, respectively) and square dance (mean 4.79, SD 1.16 mmol/L; mean 1.27, SD 0.34 mmol/L; and mean 2.18, SD 0.47 mmol/L, respectively) groups than in the control group (mean 5.79, SD 0.90 mmol/L; mean 1.93, SD 0.68 mmol/L; and mean 2.91, SD 0.77 mmol/L; and *P*=.006, *P*=.001, and *P*=.001, respectively). HDL-C was higher in the CTC12 group (mean 1.53, SD 0.30 mmol/L) than in the control group (mean 1.27, SD 0.19 mmol/L), although this did not reach statistical significance (*P*=.08).

Fasting glucose was significantly lower in the CTC12 and square dance groups than in the control group (*P*=.02), whereas IL-6 did not differ significantly among groups (*P*=.92).

SPPB and WHOQOL scores were significantly higher in the CTC12 and square dance groups than in the control group (*P*=.01 and *P*=.03, respectively), while ADL did not differ between groups (*P*=.12).

BMR, ΔHR, and additional energy expenditure differed significantly among groups (all *P*<.001). BMR was higher in the CTC12 (mean 1391.50, SD 70.78 kcal) and square dance (mean 1396.55, SD 74.45 kcal) groups than in the TC24, walking, and control groups. ΔHR was greatest in the square dance group (mean 46.68, SD 7.48 bpm), followed by the CTC12 group, which showed an increase of mean 29.50 (SD 4.61) bpm, indicating significant differences between groups (*P*<.001).

Additional energy expenditure during 1.5-h sessions was also higher in the square dance (mean 386.65, SD 37.29 kcal) and CTC12 (mean 363.75, SD 18.54 kcal) groups than in the other groups, indicating greater exercise intensity (Table 3; Figure S1 in Multimedia Appendix 3).

### Secondary Outcomes: WHOQOL Subdomains

#### Within-Group Comparisons

Following the intervention, improvements were observed across multiple WHOQOL subdomains. Q1 and Q2 scores significantly improved in all intervention groups (CTC12, square dance, TC24, and walking; all *P*<.05), whereas no significant changes were observed in the control group. In contrast, Q3 improved significantly only in the square dance group (*P*=.003), with no significant changes in the other groups. The environmental quality of life (Q4) remained unchanged across all groups (*P*>.05), although the square dance group showed a borderline decline (*P*=.05). Self-perceived health status (Q5) significantly improved in all intervention groups (*P*<.01). Similarly, self-rated overall quality of life (Q6) increased significantly in all intervention groups (*P*<.01), with no change observed in the control group (Table S1 in Multimedia Appendix 3).

### Between-Group Comparisons

Postintervention comparisons showed varying patterns across subdomains. Q1 scores were highest in the square dance group and lowest in the control group, with a marginal group effect (*P*=.06). Q2 scores were significantly higher in the CTC12 and square dance groups than in the control group (*P*=.005), indicating improved psychological health. No significant differences were observed for Q3 (*P*=.13), although the square dance group had the highest mean score. Similarly, no significant between-group differences were observed for Q4 (*P*=.78). For Q5, a near-significant group effect was observed (*P*=.09), with higher scores in the CTC12 and square dance groups than in the control group. Q6 did not differ significantly among groups (*P*=.13), although the CTC12 group showed the highest mean overall quality of life score (Table S2 and Figure S2 in Multimedia Appendix 3).

## Discussion

### Overview of Intervention Effects

This 12-week exercise intervention demonstrated significant benefits in the CTC12 and square dance groups. Both interventions improved lipid profiles, with TC reduced in the square dance group and triglyceride and LDL-C reduced in both the CTC12 and square dance groups, while no significant changes were observed in FPG or IL-6. Functional outcomes also improved, as reflected by increased SPPB scores in both groups and improved ADL scores in the square dance group. WHOQOL scores significantly improved in the CTC12, square dance, and walking groups, indicating enhanced quality of life. Collectively, these findings support the effectiveness of CTC12 and square dance in improving lipid metabolism, physical function, and quality of life in middle-aged and older adults.

### Improvements in Cardiometabolic Indicators and Underlying Mechanisms

Regarding blood pressure, although no significant changes were observed in DBP, SBP, or PP across the groups, small trends in blood pressure changes were noted. From a clinical perspective, such small blood pressure changes are unlikely to represent meaningful short-term cardiovascular risk reduction, although minor population-level shifts have been associated with long-term risk trends in epidemiological studies [65]. Mechanistically, CTC12 may influence SBP through coordinated breathing and postural control, potentially modulating autonomic balance and arterial compliance [48]. The square dance intervention, characterized by rhythmic and moderate-to-high intensity movements, may contribute to improvements in large-artery elasticity, which could partly explain the observed PP reduction [66]. Walking, as a low-intensity aerobic activity, may enhance endothelial function and peripheral vasodilation, contributing to modest DBP changes [67]. These mechanisms provide plausible explanations for the observed directional trends but do not imply clinically significant blood pressure reductions within the intervention period of this study.

The CTC12 and square dance groups demonstrated significant reductions in TC, triglyceride, and LDL-C, while HDL-C was significantly increased in the CTC12 group, indicating beneficial effects on lipid metabolism. The CTC12 regimen, which incorporates strength, coordination, and breathing control, may enhance lipid homeostasis and contribute to increases in HDL-C [68]. In contrast, square dance may improve lipid profiles through enhanced lipoprotein lipase activity, facilitating very-low-density lipoprotein clearance and cholesterol transport [68]. Although the CTC12 group exhibited greater increases in HDL-C, and the square dance group showed more pronounced reductions in TC, both groups demonstrated complementary effects on lipid regulation, which may have beneficial implications for cardiovascular prevention [69]. The square dance group exhibited a more pronounced reduction in TC, whereas the CTC12 group was more effective in elevating HDL-C, indicating distinct yet complementary lipid-regulatory mechanisms. Both groups achieved clinically relevant lipid thresholds (eg, triglyceride<1.7 mmol/L and LDL-C<3.4 mmol/L), underscoring their potential role in primary cardiovascular prevention. This suggests that the observed lipid improvements were not only statistically significant but also potentially relevant for practical cardiovascular risk management in community-dwelling older adults [70].

Although not statistically significant, the control group exhibited a worsening trend in TC, triglyceride, and other cardiometabolic markers, which may reflect the absence of structured physical activity [71].

### Modulation of Inflammatory Markers

No significant changes in IL-6 levels were observed across intervention groups. This may reflect the relatively healthy baseline status of the participants and the limited duration of the intervention. In such populations, longer intervention periods may be required to detect measurable changes in inflammatory markers. Although mind–body interventions, such as CTC12 and Square Dance, may influence parasympathetic activity and low-grade inflammation, these effects did not translate into detectable changes in IL-6 within the short intervention period. Future studies with extended follow-up are needed to clarify these effects [72].

### Improvements in Physical Function and BMR

The CTC12 group demonstrated the greatest improvements in SPPB scores, followed by the square dance group, whereas no significant changes were observed in the walking group. The coordinated activation of core and lower-limb musculature in CTC12 likely contributes to improvements in balance, coordination, and postural control, thereby reducing the risk of falls and improving gait performance. These improvements may be clinically meaningful, as better SPPB performance is closely associated with mobility, reduced fall risk, and preserved functional independence in older adults [73].

Square dance, characterized by rhythmic movement and group participation, was associated with increases in BMR, suggesting enhanced metabolic activity and energy efficiency, which may help delay age-related metabolic decline [74].

### Improvements in Quality of Life and Mental Health

WHOQOL scores improved in the CTC12, square dance, and TC24 groups, with the most pronounced improvements in psychological health gains (Q2) observed in the CTC12 and square dance groups. The underlying mechanisms appear distinct. CTC12 may enhance emotional regulation and self-efficacy through long-term cognitive engagement and controlled breathing, facilitating parasympathetic activation [75]. Conversely, the square dance may improve mood, social cohesion, and subjective well-being through social interaction, rhythmic movement, and music [76]. These findings suggest that CTC12 emphasizes internal self-regulation, whereas square dance operates through external social reinforcement, highlighting the importance of tailoring interventions to individual psychological and social profiles [63]. A slight worsening trend in psychological scores was observed in the control group, which may reflect the effects of prolonged inactivity [77].

### Safety and Feasibility of the Interventions

Mean exercise HR across all intervention groups remained within 50%-70% of HR_max, consistent with American College of Sports Medicine guidelines for middle-aged and older adults [78]. No falls or cardiovascular events were observed in the CTC12 group, despite the inclusion of explosive movements, likely due to structured warm-up protocols, progressive difficulty, and professional supervision. Similarly, no injuries were reported in the square dance group, despite engaging in moderate-to-high intensity activities. Attendance exceeded 90% across all groups, indicating high adherence and feasibility. These findings support the safety and applicability of these interventions in community-based settings.

### Dose-Related Interpretation and Practical Considerations

A descriptive dose-related interpretation was used to examine how differences in exercise intensity and physiological load relate to the breadth of observed health benefits. CTC12 and square dance improved a greater number of outcomes and were therefore compared in terms of exercise dose characteristics and physiological demand. Square dance demonstrated higher energy expenditure (386.7 kcal) and ΔHR (46.7 bpm; approximately 72%-75% HR_max), reflecting moderate-to-high intensity activity. This higher intensity may explain the more rapid improvements in physical function but may also exceed optimal safety thresholds for some older adults, particularly those with cardiovascular risk factors, potentially limiting long-term sustainability. Nevertheless, square dance led to more immediate improvements in physical function, particularly in balance and muscular endurance, possibly due to its higher energy expenditure [79]. CTC12 achieved relatively high energy expenditure (363.8 kcal) and metabolic activation while maintaining a lower cardiovascular load (ΔHR=29.5 bpm; approximately 60%-65% HR_max), consistent with recommended safety ranges for older adults [80]. Although improvements appeared more gradual, CTC12 was associated with long-term benefits across physical, psychological, and quality-of-life domains with a lower risk of excessive fatigue or cardiovascular strain. These findings suggest distinct practical roles for each intervention. Square dance may be more suitable for individuals seeking higher-intensity activity and faster functional gains, whereas CTC12 may provide a more sustainable, lower-risk option for long-term health maintenance [81-83]. From an implementation perspective, both interventions are feasible in community settings; however, CTC12 may be more appropriate for broader populations due to its moderate intensity and lower cardiovascular demand, whereas square dance may require closer monitoring in higher-risk individuals [84,85].

### Limitations

This study has some limitations. The relatively small sample size (n=22 per group) may have limited statistical power, and the 12-week intervention period may not have been adequate to capture long-term effects. Although outcome assessors were blinded to group allocation, participants were necessarily aware of their assigned intervention due to the distinct characteristics of each exercise modality. This awareness may have introduced expectancy-related influences, particularly for self-reported outcomes such as quality of life (eg, WHOQOL). However, such effects are less likely to have substantially influenced objective measures, including blood pressure, biochemical indicators, and physical function. Laboratory personnel responsible for biochemical analyses were blinded to group assignment, which helped mitigate detection bias.

Although the final sample size was adequate for the primary group×time analysis, the study may have been underpowered to detect differences in some secondary outcomes. Accordingly, these findings should be interpreted with caution. In addition, advanced cardiovascular assessments were not included, which limits the ability to characterize underlying physiological changes in greater detail. Future studies should include larger and more diverse samples, extend intervention duration, and incorporate participants at elevated clinical risk. The application of advanced analytical approaches, such as structural equation modeling, may facilitate a more comprehensive understanding of the interrelated metabolic, neural, emotional, and social pathways influenced by exercise. Building on the findings of this study in relatively healthy middle-aged and older adults, further research in populations with cardiovascular disease, metabolic disorders, and other chronic conditions is warranted to clarify clinical benefits and underlying mechanisms across different disease contexts.

### Conclusion

In summary, we demonstrated that both CTC12 and square dance were associated with improvements in blood lipid profiles, physical function, and quality of life in middle-aged and older adults. CTC12 was associated with improvements across multiple health indicators, whereas square dance showed more pronounced effects on physical function, metabolic activity, and emotional well-being. In contrast, the effects observed for TC24 and walking were more limited and were confined to selected outcomes. These findings support the role of structured, moderate-to-high-intensity exercise in promoting healthy aging and provide preliminary evidence to inform the development of targeted exercise interventions for age-related health conditions.

## Appendix

Multimedia Appendix 1 Additional figures.

Multimedia Appendix 2 CONSORT-eHEALTH checklist (V 1.6.1).

Multimedia Appendix 3 Additional Figures and Tables.

## Abbreviations

ΔHR: change in heart rate
ADL: activity of daily living
BMR: Basal metabolic rate
CONSORT: Consolidated Standards of Reporting Trials
CTC12: 12-form Chen-style Tai Chi
DBP: diastolic blood pressure
DM: diabetes mellitus
FPG: fasting plasma glucose
HDL-C: high-density lipoprotein cholesterol
HR: heart rate
IL-6: interleukin-6
LDL-C: low-density lipoprotein cholesterol
PP: pulse pressure
SBP: systolic blood pressure
SPPB: Short Physical Performance Battery
TC: total cholesterol
TC24: 24-form Tai Chi
WHOQOL: World Health Organization Quality of Life
