# Supplementary material for: Dose-Related Effects of Different Tai Chi Styles Versus Traditional Community-Based Exercises on Cardiometabolic Health and Physical Function in Middle-Aged and Older Adults: Randomized Controlled Trial
Source: JMIR Aging. 2026 Apr 23;9:e80125. doi: 10.2196/80125 (PMC13153752; doi:10.2196/80125)

1）.12-form Chen-style Tai Chi (CTC12)

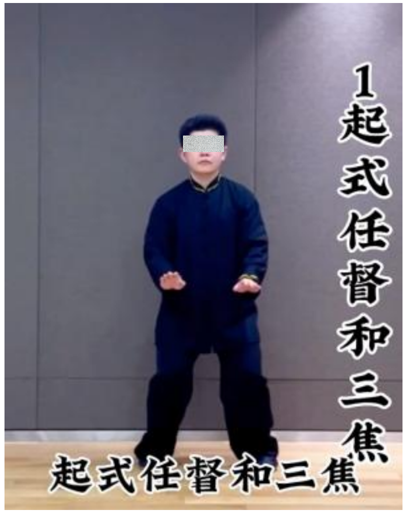

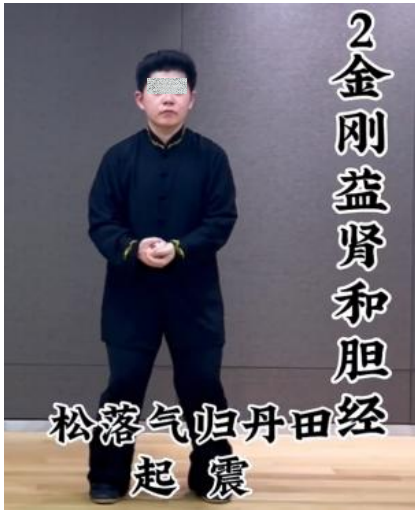

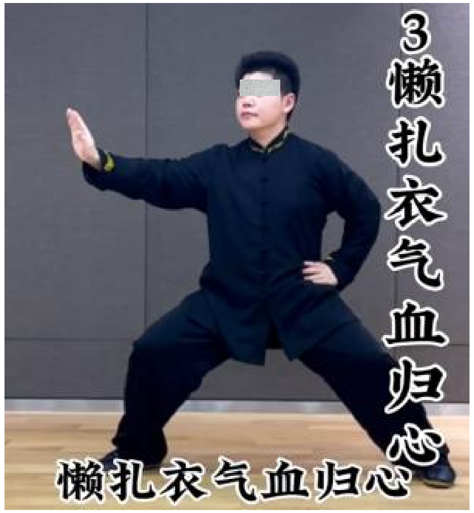

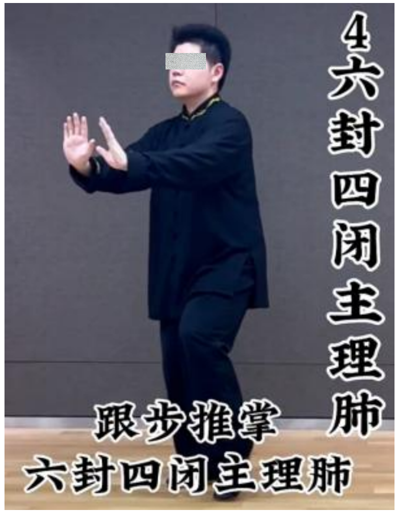

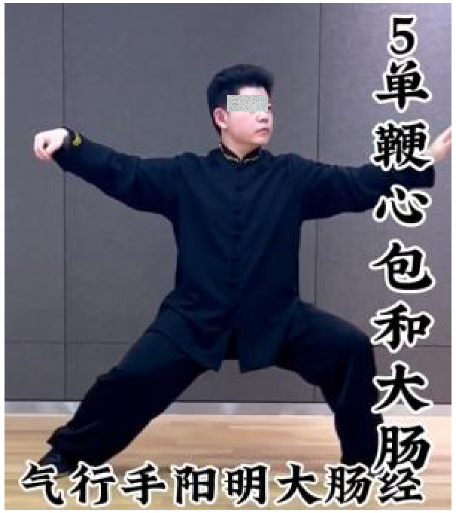

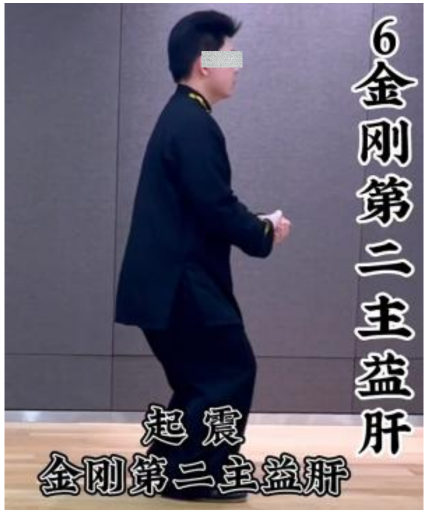

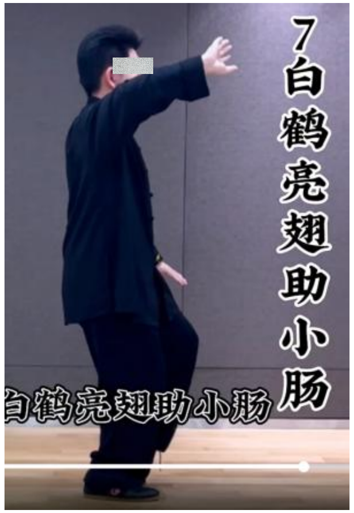

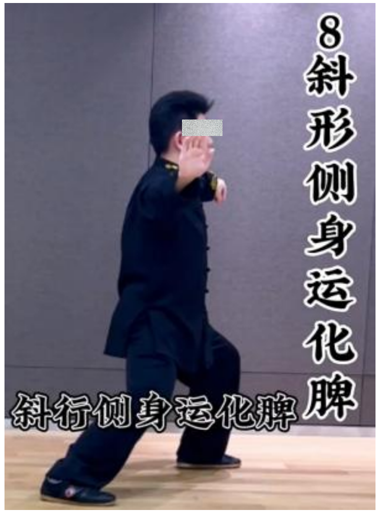

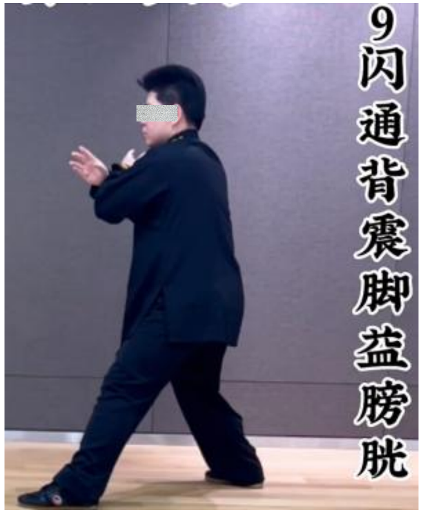

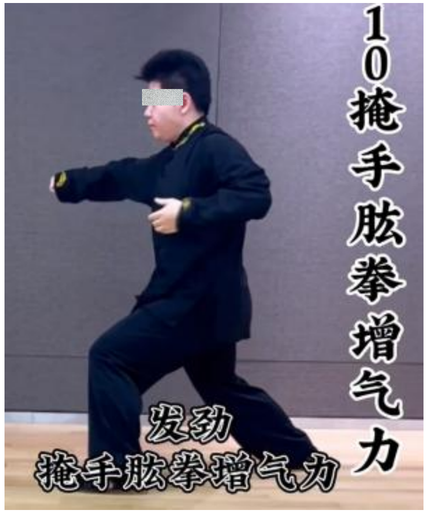

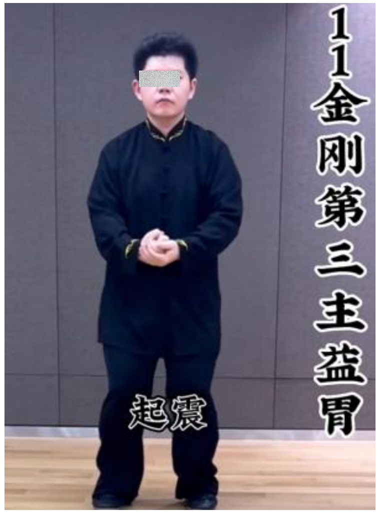

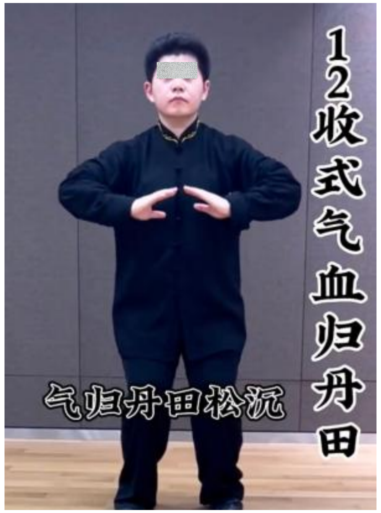

2).24-Form Tai Chi （TC24）

1、起 式

Beginning Form

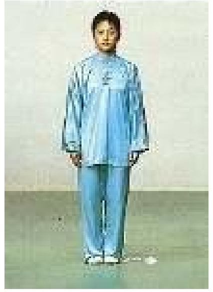
1

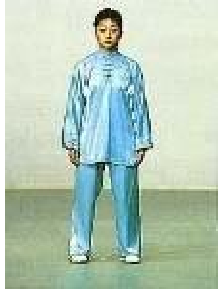
2

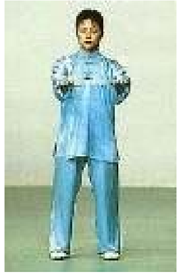
3

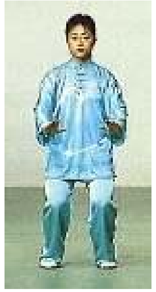
4

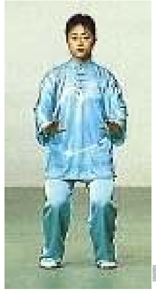
5

2、野马分鬃

Parting the Wild Horse's Mane

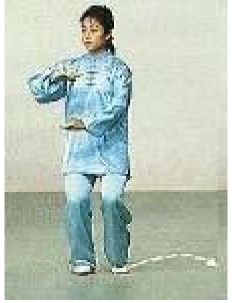

6

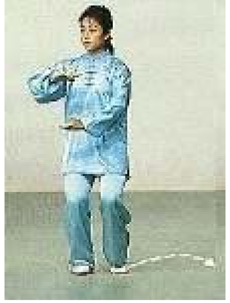

7

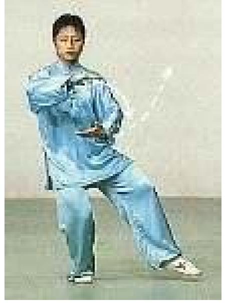

8

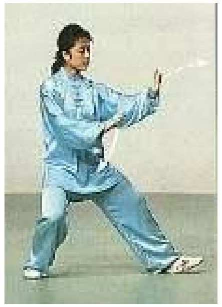

9

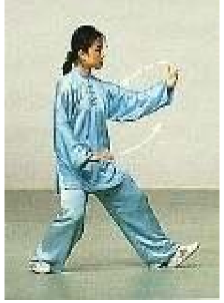

10

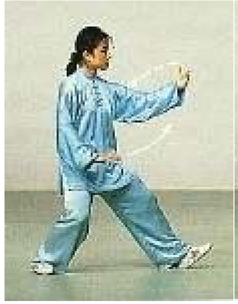

11

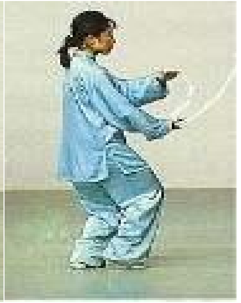

12

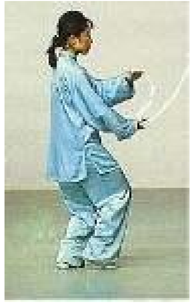

13

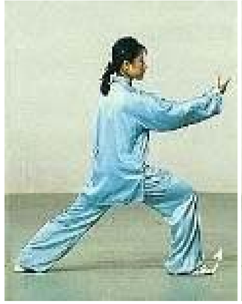

14

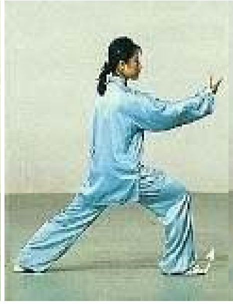

15

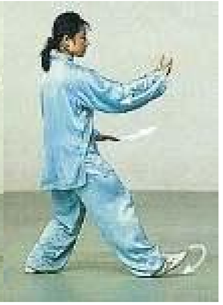

16

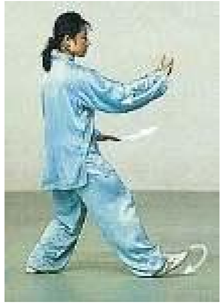

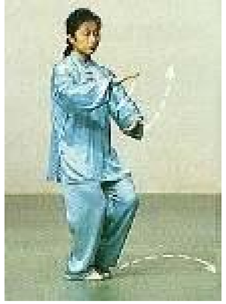

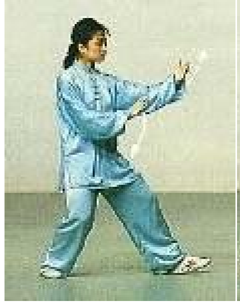

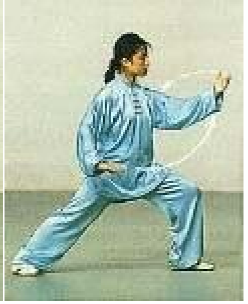

17 18 19 20

3、**白鹤亮翅**

White Crane Spreads Wings

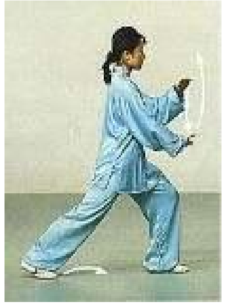

21

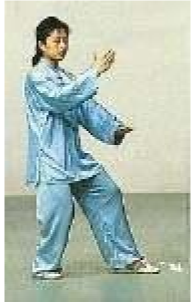

22

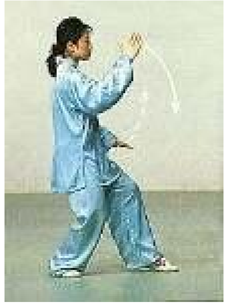

23

4、**左右搂膝拗步**

Brush Knee and Twist Step

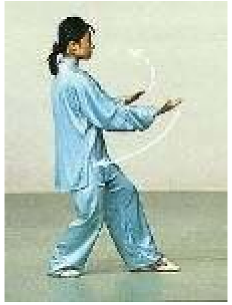

24

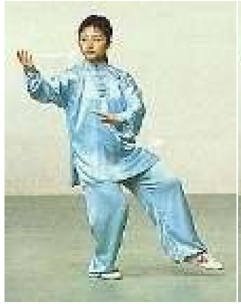

25

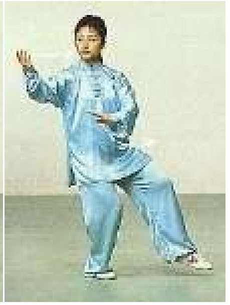

26

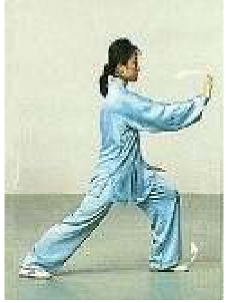

27

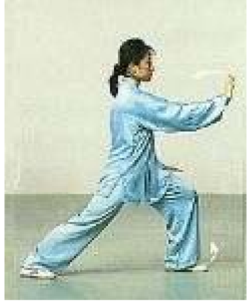

28

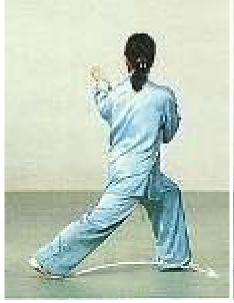

29

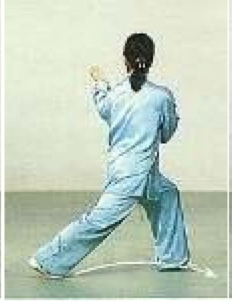

30

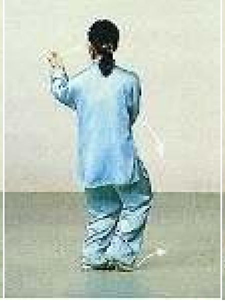

31

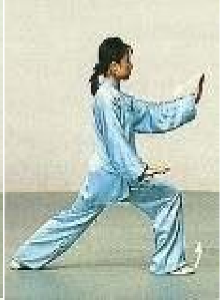

32

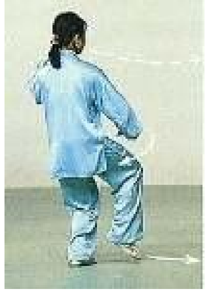

33

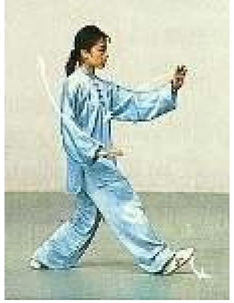

34

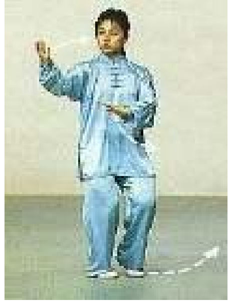

35

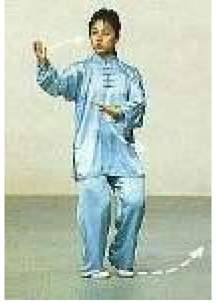

36

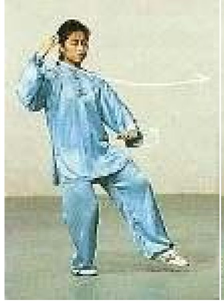

37

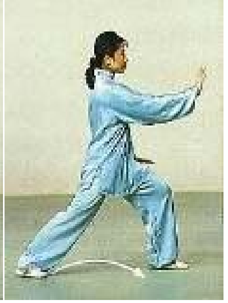

38

5、手挥琵琶

Play the Lute

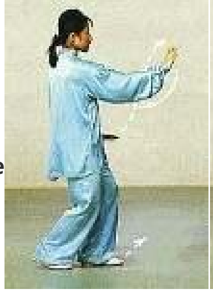

39

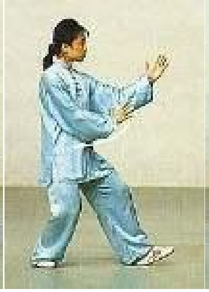

40

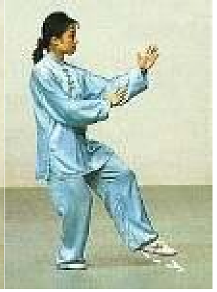

41

6、左右倒卷肱（倒撵猴 ）

Left and Right Brush Knee and Push（Repulse Monkey)

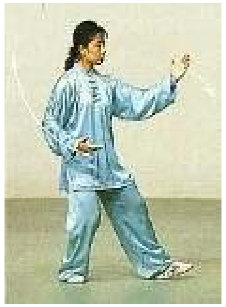

42

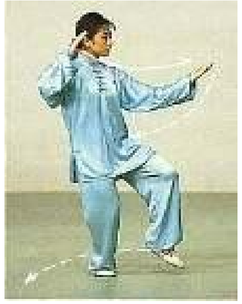

43

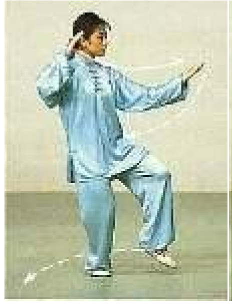

44

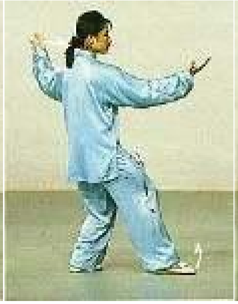

45

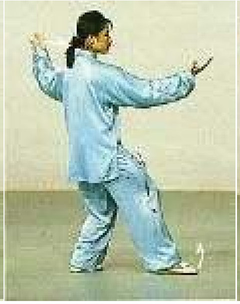

46

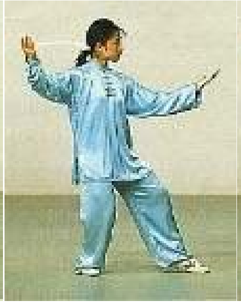

47

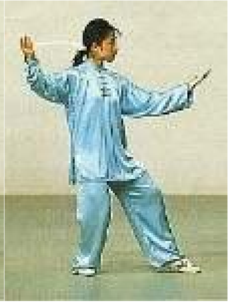

48

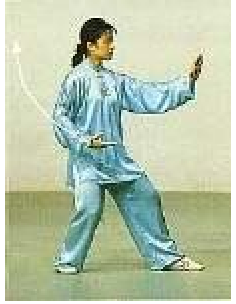

49

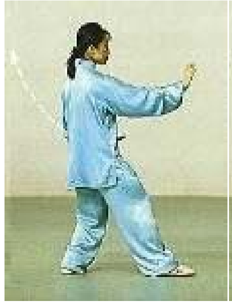

50

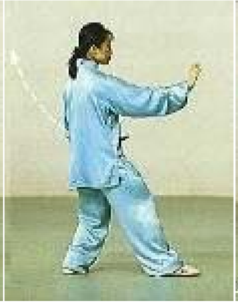

51

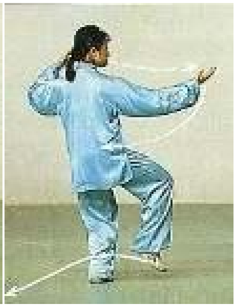

52

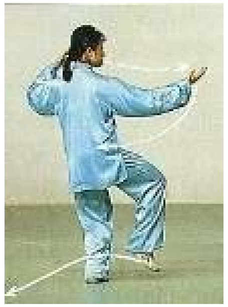

53

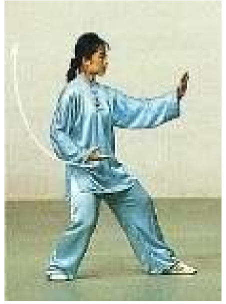

54

7、左拦雀尾

Grasp the Bird's Tail on the Left

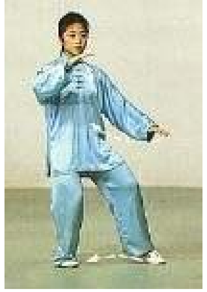
55

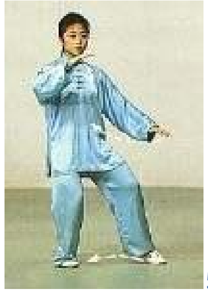
56

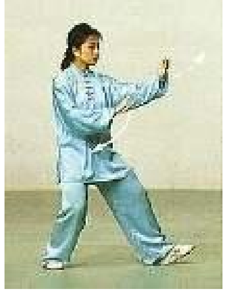
57

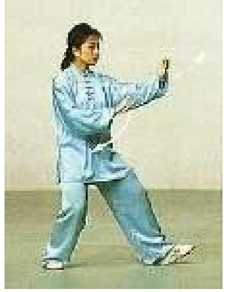
58

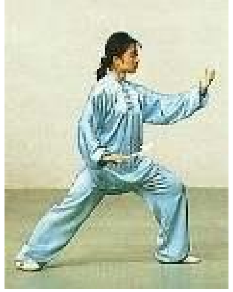
59

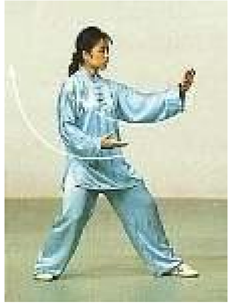
60

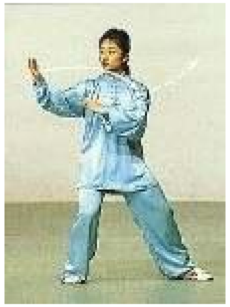
61

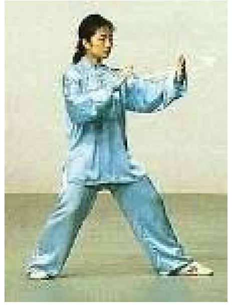
62

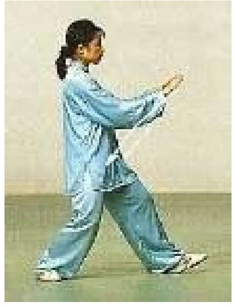
63

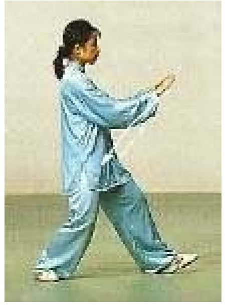
64

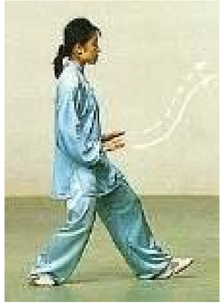
65

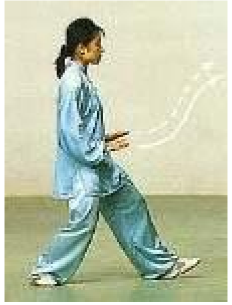
66

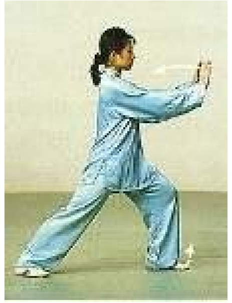
67

8、右拦雀尾

Grasp the Bird's Tail on the Right

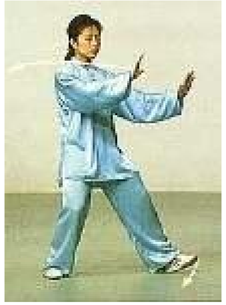
68

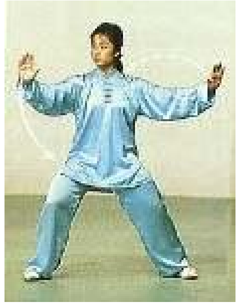
69

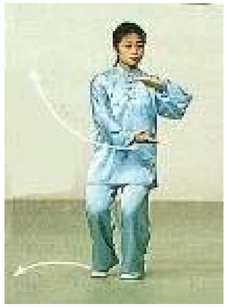
70

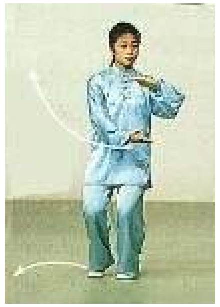

71

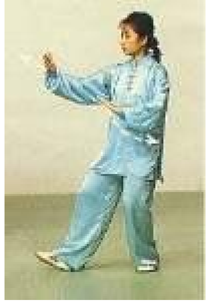

72

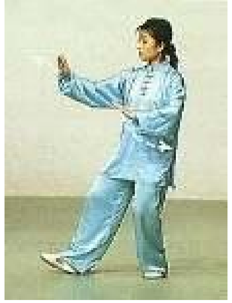

73

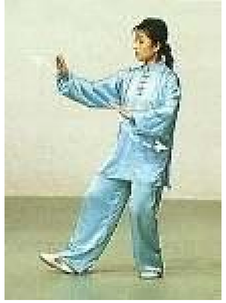

74

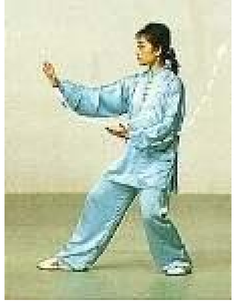

75

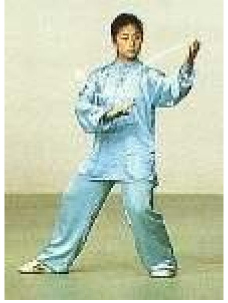

76

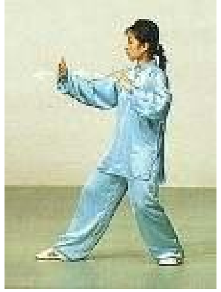

77

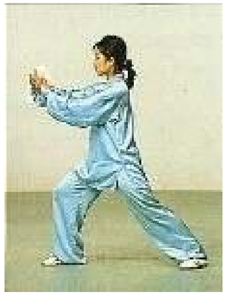

78

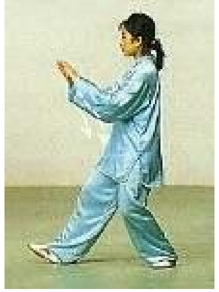

79

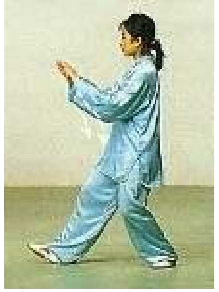

80

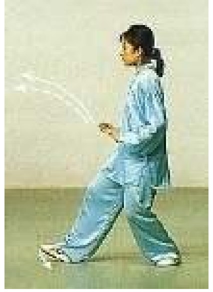

81

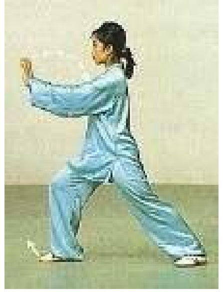

82

9、单 鞭

Single Whip

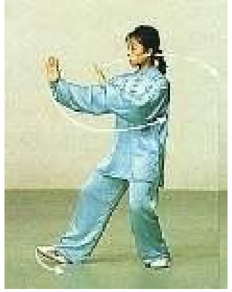

83

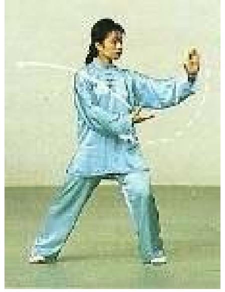

84

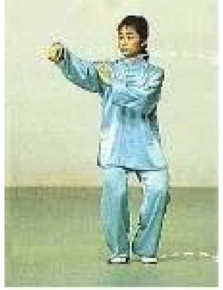

85

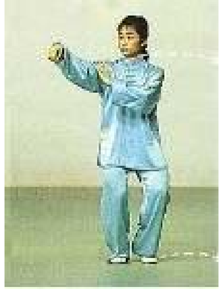

86

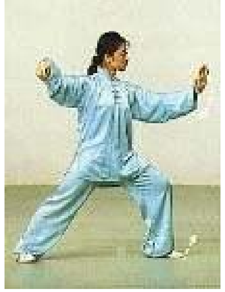

87

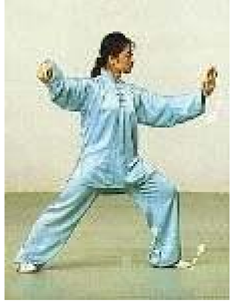

88

10、云 手

Wave Hands Like Clouds

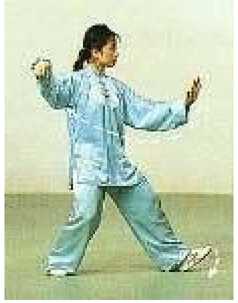

89

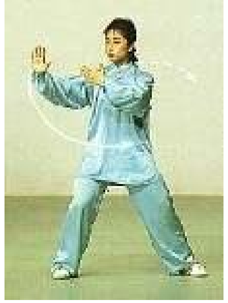

90

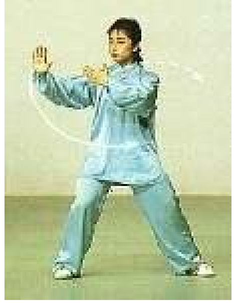

91

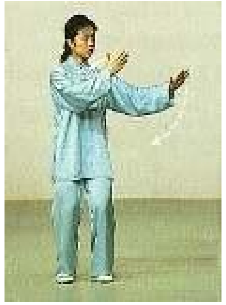

92

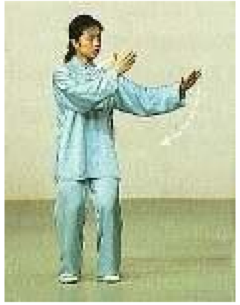

93

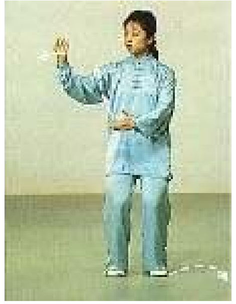

94

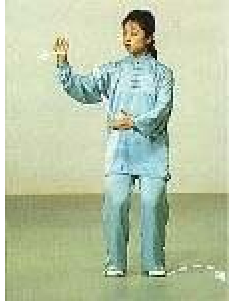

95

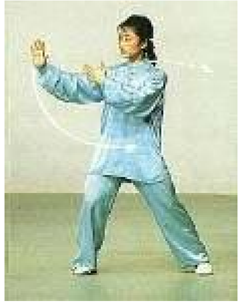

96

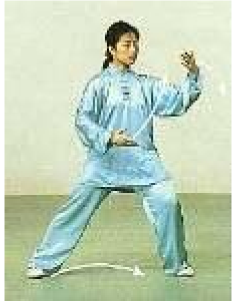

97

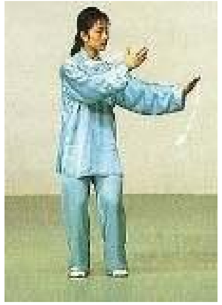

98

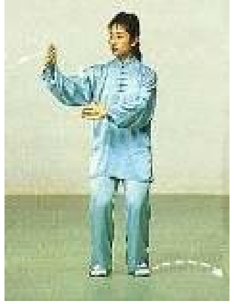

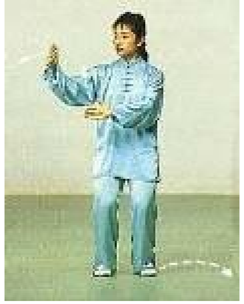

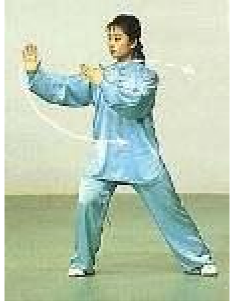

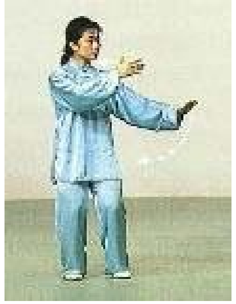

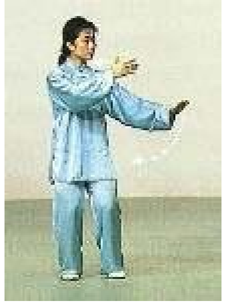

11、单 鞭

Single Whip

99 100 101 102 103

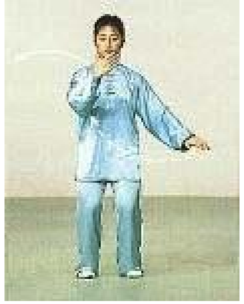

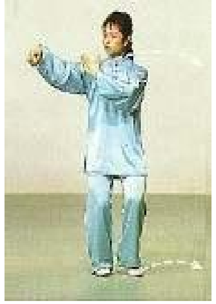

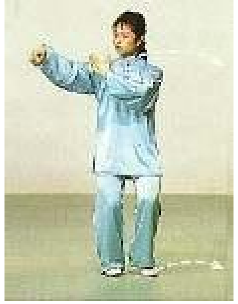

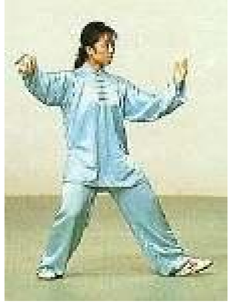

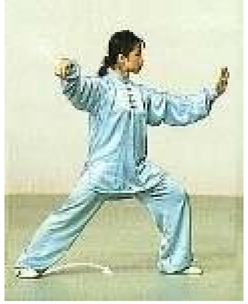

12、高探马

High Pat on Horse

104 105 106 107 108

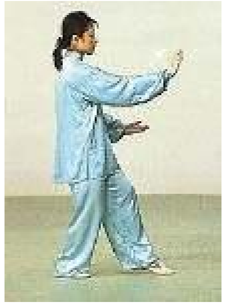

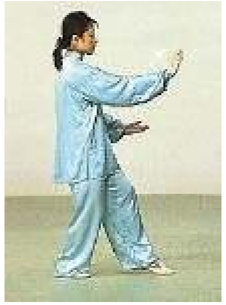

13、右蹬脚

Kick with Right Heel

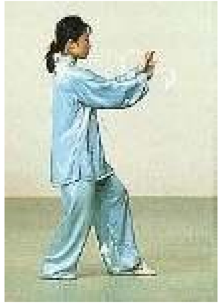

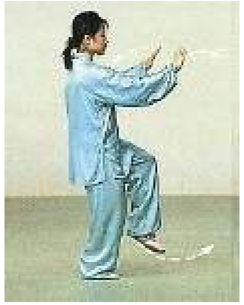

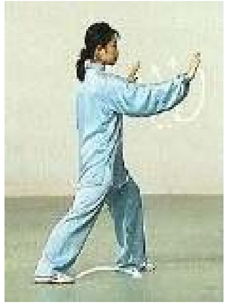

109 110 111 112 113

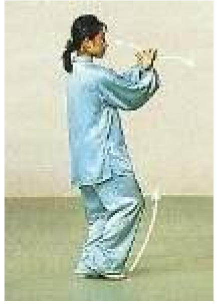

114

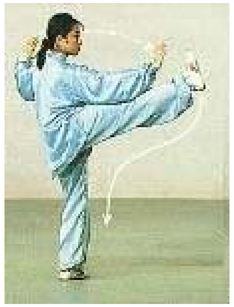

115

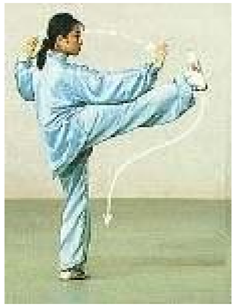

116

14、双峰贯耳

Strike to Ears with Both Fists)

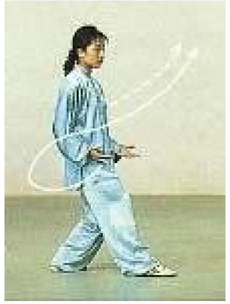

117

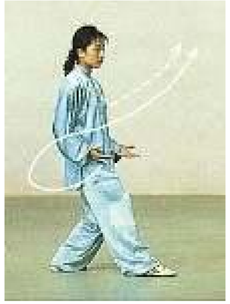

118

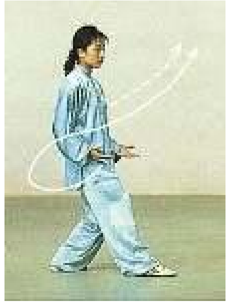

119

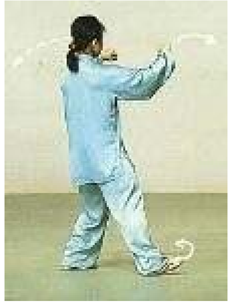

120

15、转身左蹬脚

Turn Body and Kick with Left Heel

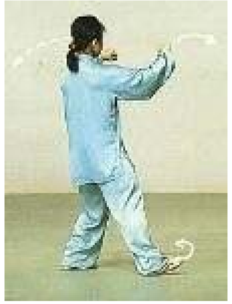

121

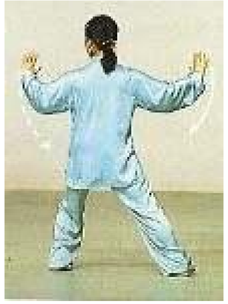

122

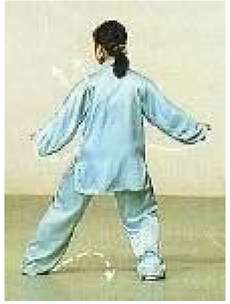

123

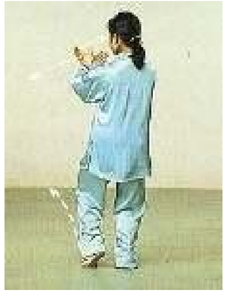

124

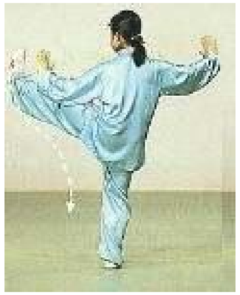

125

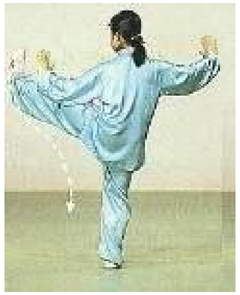

126

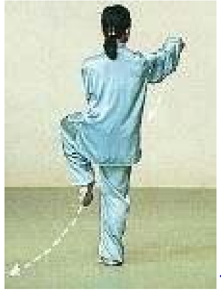

127

16、左下势独立
Left Lower Body and Stand on One Leg

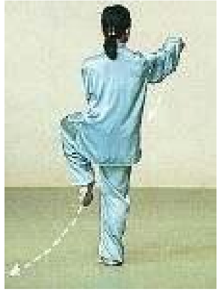
128

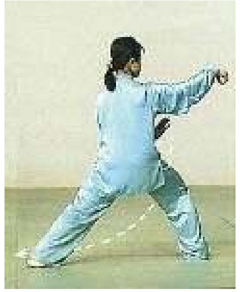
129

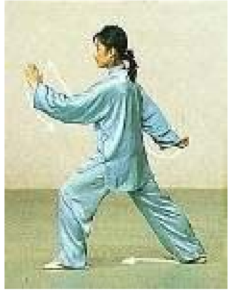
130

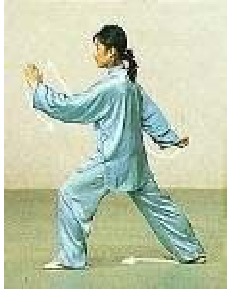
131

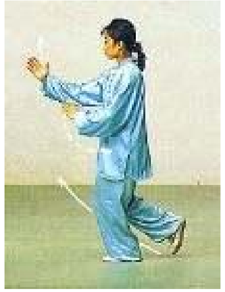
132

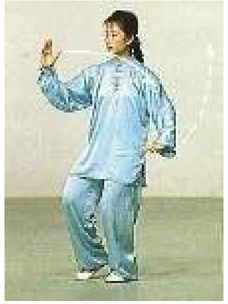
133

17、右下势独立
Right Lower Body and Stand on One Leg

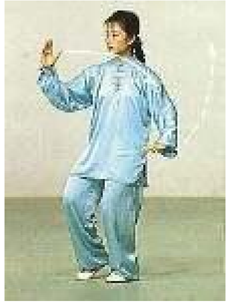
134

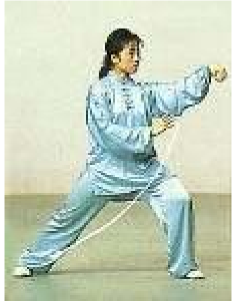
135

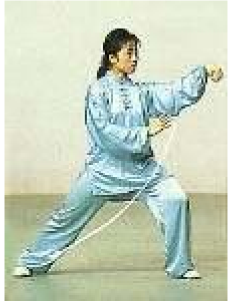
136

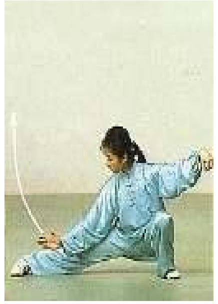
137

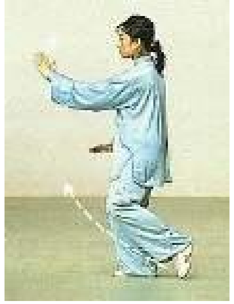

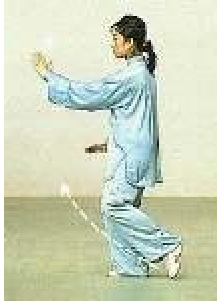

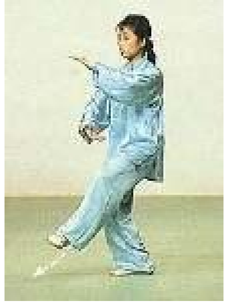

18、右左穿梭
Fair Lady Works Shuttles

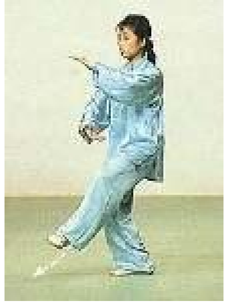

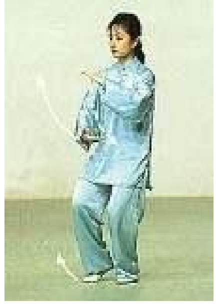

138 139 140 141 142

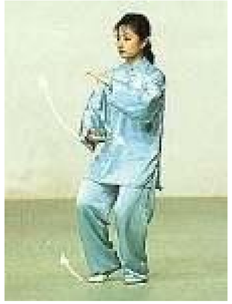
143

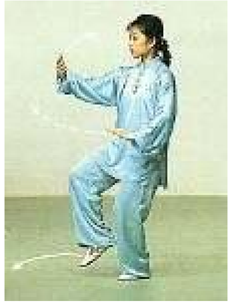
144

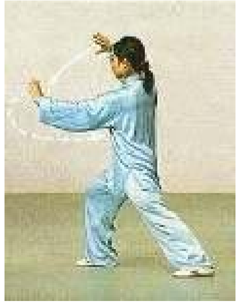
145

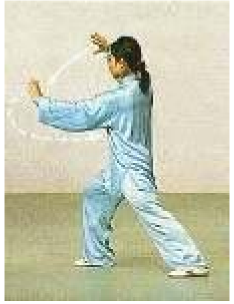
146

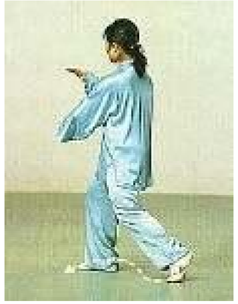
147

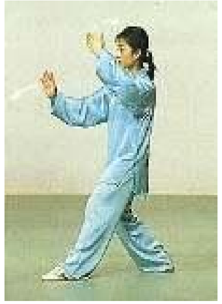
148

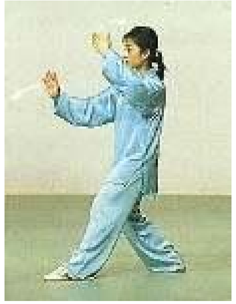
149

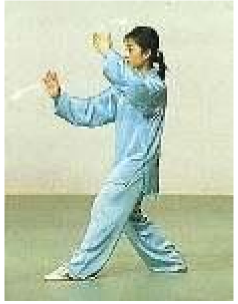
150

19、**海底针 Needle at Sea Bottom**

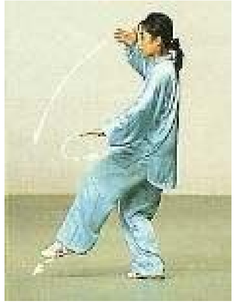
151

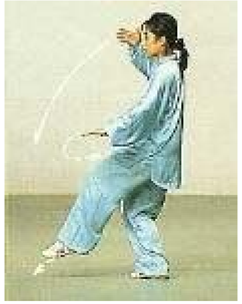
152

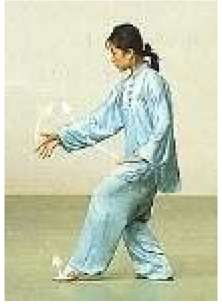
153

20、闪通臂

Fan Through Back

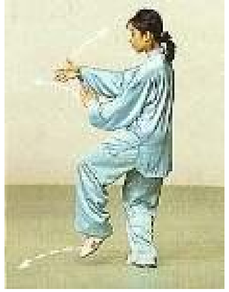

154

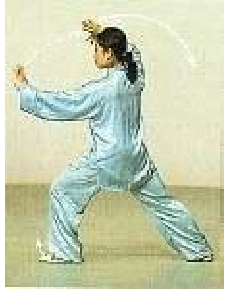

155

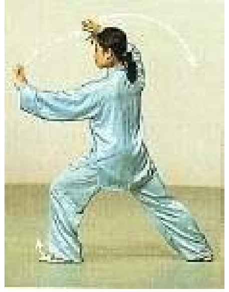

156

21、转身搬拦锤

Turn Body and Chop with Fist)

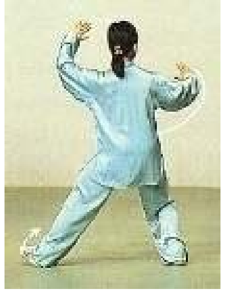

157

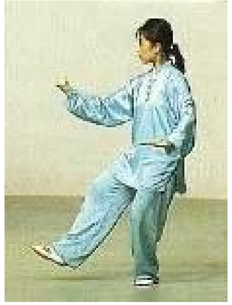

160

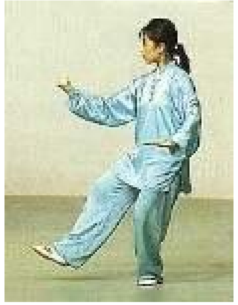

161

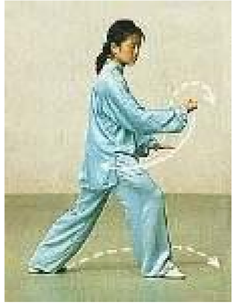

162

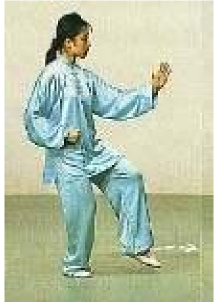

163

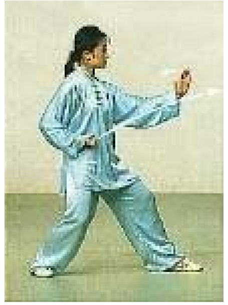

164

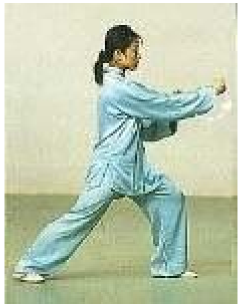

165

22、如封似闭

Apparent Closing Up

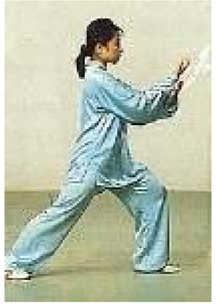

166

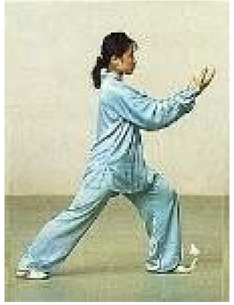

167

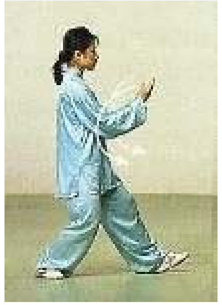

168

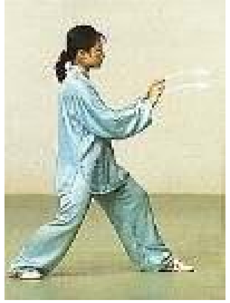

169

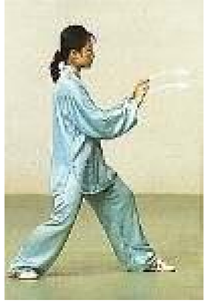

170

23、十字手

Cross Hands

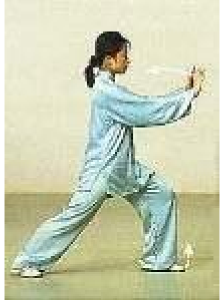

171

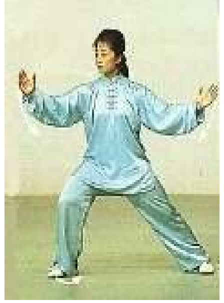

172

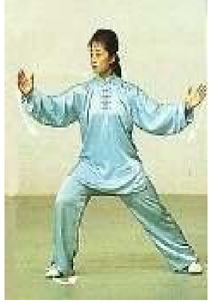

173

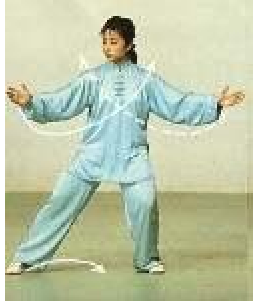

174

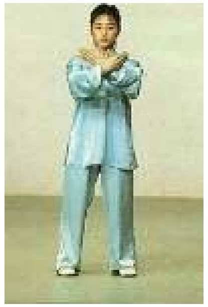

175

24、收 势

Closing Form

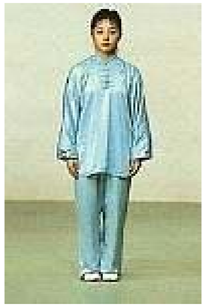

178

3).Square Dance

1 垫步左右伸展” (Diànbù Zuǒyòu Shēnzhǎn)

"Step and Reach Left and Right"

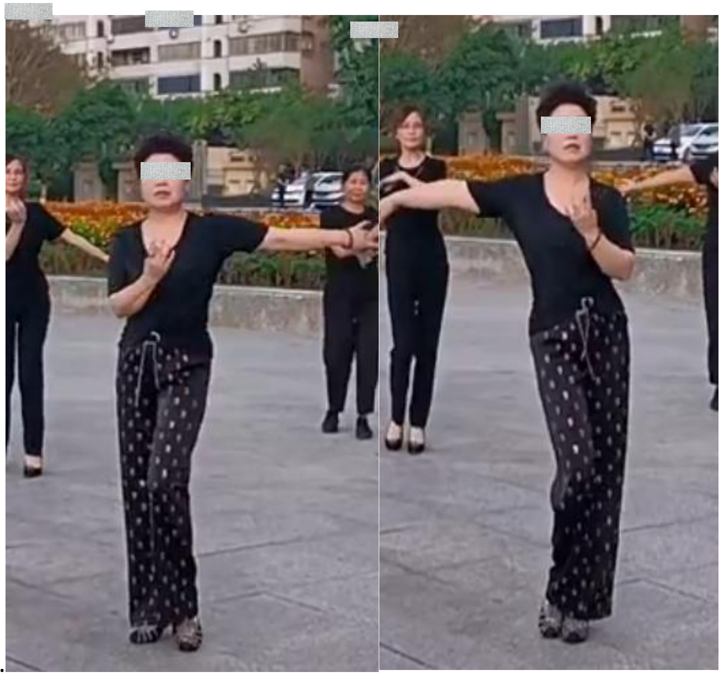

.

2.垫步向上伸展” (Diànbù Xiàng Shàng Shēnzhǎn)

"Step and Reach Upward"

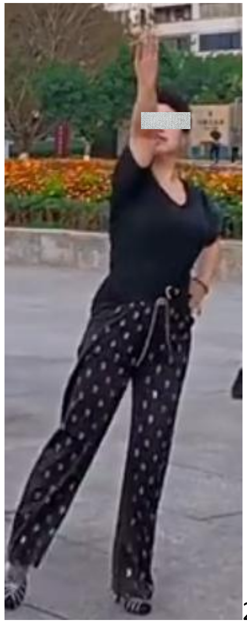2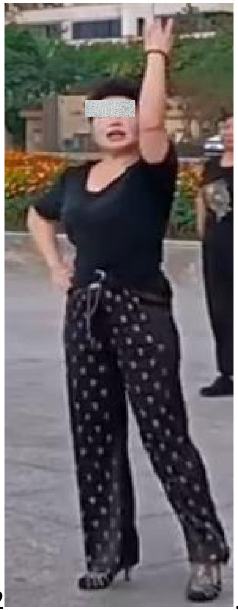.

3 曲臂垫步左右转髋” (Qū bì Diànbù Zuǒyòu Zhuǎn kuāi)
"Bent Arm Step and Rotate Hips Left and Right"

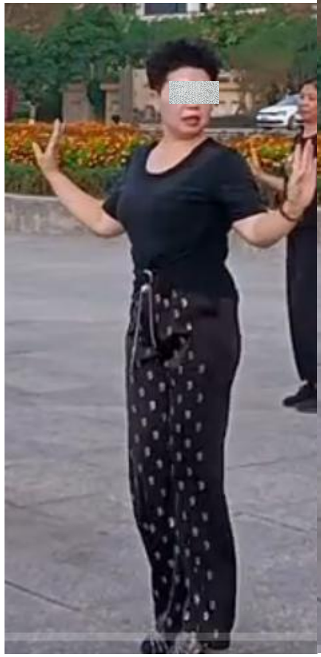
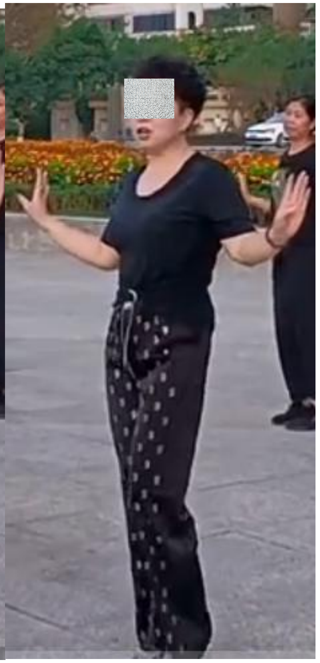

4.垫步向上伸展” (Diànbù Xiàng Shàng Shēnzhǎn)

"Step and Reach Upward"

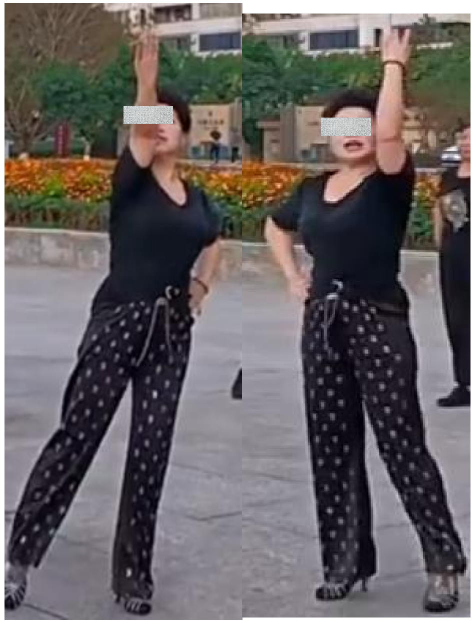

5.曲臂垫步向前伸展” (Qū bì Diànbù Xiàng Qián Shēnzhǎn)
"Bent Arm Step and Reach Forward"

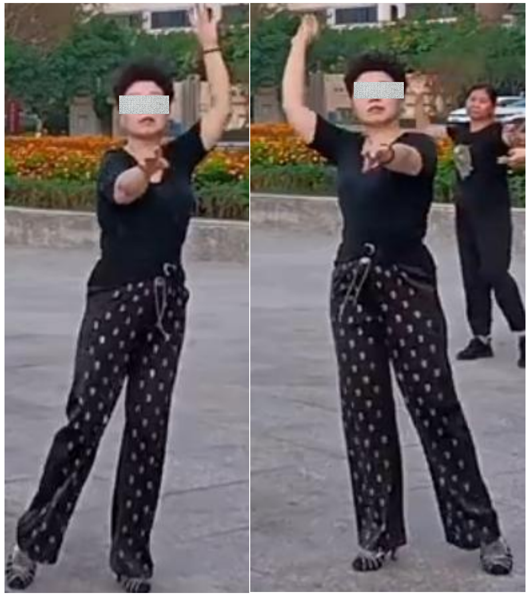

Supplement: Multimedia Appendix 1 [file aging_v9i1e80125_app1.pdf]
