# Supplementary material for: Dose-Related Effects of Different Tai Chi Styles Versus Traditional Community-Based Exercises on Cardiometabolic Health and Physical Function in Middle-Aged and Older Adults: Randomized Controlled Trial
Source: JMIR Aging. 2026 Apr 23;9:e80125. doi: 10.2196/80125 (PMC13153752; doi:10.2196/80125)
Supplement: Multimedia Appendix 3 [file aging_v9i1e80125_app3.docx]

**Table S1. Within-group comparisons of WHOQOL subdomains before and after intervention (paired *t* tests).**

| **Variable** | **Group** | **Pre-**  **intervention**  **（Mean±SD）** | **Post-**  **intervention**  **（Mean±SD）** | **t-value** | **p-value** | **Change** | |
| --- | --- | --- | --- | --- | --- | --- | --- |
|  |  |  |  |  |  | **Marginal means** | **95% confidence interval** |
| **WHOQOL Q1 Physical Health (scores)** | CTC12 | 26.91 ± 1.97 | 27.50 ± 1.74 | 2.8897 | **.009** | 0.59 | 0.17 to 1.02 |
|  | TC24 | 26.96 ± 1.85 | 27.39 ± 1.56 | 2.4721 | **.022** | 0.43 | 0.07 to 0.80 |
|  | Square Dance | 26.77 ± 3.05 | 28.00 ± 2.49 | 3.3539 | **.003** | 1.23 | 0.48 to 1.99 |
|  | Walking | 26.96 ± 2.69 | 27.39 ± 2.39 | 2.5543 | **.018** | 0.43 | 0.09 to 0.87 |
|  | Control | 27.00 ± 2.07 | 26.57 ± 1.78 | 1.686 | .106 | -0.43 | -0.97 to 0.10 |
| **WHOQOL Q2 Psychological Health (scores)** | CTC12 | 24.78 ± 2.02 | 26.14 ± 1.98 | 4.2712 | **<.001** | 1.36 | 0.70 to 2.03 |
|  | TC24 | 24.82 ± 1.61 | 25.78 ± 1.44 | 2.5911 | **.017** | 0.96 | 0.19 to 1.72 |
|  | Square Dance | 24.81 ± 1.79 | 25.91 ± 1.44 | 4.2941 | **<.001** | 1.10 | 0.56 to 1.62 |
|  | Walking | 24.78 ± 2.19 | 25.35 ± 1.80 | 2.8680 | **.009** | 0.57 | 0.16 to 0.97 |
|  | Control | 24.83 ± 2.01 | 24.44 ± 1.67 | -1.8166 | .083 | -0.39 | -0.84 to 0.06 |
| **WHOQOL Q3 Social Relationships (scores)** | CTC12 | 11.64 ± 1.33 | 11.77 ± 1.41 | 0.5684 | .576 | 0.13 | -0.36 to 0.64 |
|  | TC24 | 11.61 ± 1.37 | 11.65 ± 1.23 | 0.2246 | .824 | 0.04 | -0.36 to 0.44 |
|  | Square Dance | 11.64 ± 1.18 | 12.27 ± 1.03 | 3.3086 | **.003** | 0.63 | 0.24 to 1.04 |
|  | Walking | 11.65 ± 1.34 | 11.83 ± 1.19 | 1.0000 | .328 | 0.18 | -0.19 to 0.53 |
|  | Control | 11.61 ± 1.50 | 11.30 ± 1.22 | -1.9084 | .070 | -0.31 | -0.64 to 0.03 |
| **WHOQOL Q4 Environment (scores)** | CTC12 | 31.55 ± 3.89 | 31.45 ± 3.31 | -0.3265 | .747 | -0.10 | -0.67 to 0.49 |
|  | TC24 | 31.35 ± 3.60 | 31.65 ± 2.60 | 0.4375 | .666 | 0.30 | -1.14 to 1.75 |
|  | Square Dance | 31.32 ± 3.46 | 30.55 ± 3.86 | -2.0784 | .050 | -0.77 | -1.55 to 0.00 |
|  | Walking | 30.96 ± 3.86 | 30.78 ± 3.72 | -0.5462 | .590 | -0.18 | -0.83 to 0.49 |
|  | Control | 31.09 ± 3.00 | 31.48 ± 3.22 | 1.8993 | .071 | 0.39 | -0.04 to 0.82 |
| **WHOQOL Q5 Self-Perceived Health (scores)** | CTC12 | 3.55 ± 0.51 | 3.95 ± 0.58 | 3.8129 | **.001** | 0.40 | 0.19 to 0.63 |
|  | TC24 | 3.57 ± 0.51 | 3.87 ± 0.55 | 3.1024 | **.005** | 0.30 | 0.10 to 0.51 |
|  | Square Dance | 3.64 ± 0.49 | 4.00 ± 0.44 | 3.4641 | **.002** | 0.36 | 0.15 to 0.58 |
|  | Walking | 3.65 ± 0.49 | 3.91 ± 0.67 | 2.7865 | **.011** | 0.26 | 0.07 to 0.46 |
|  | Control | 3.65 ± 0.49 | 3.57 ± 0.59 | -0.4917 | .628 | -0.08 | -0.45 to 0.28 |
| **WHOQOL Q6 Self-rated Overall Quality of Life (scores)** | CTC12 | 3.95 ± 0.58 | 4.41 ± 0.59 | 3.5784 | **.002** | 0.46 | 0.19 to 0.72 |
|  | TC24 | 3.96 ± 0.56 | 4.35 ± 0.57 | 3.2188 | **.004** | 0.39 | 0.14 to 0.64 |
|  | Square Dance | 3.95 ± 0.58 | 4.36 ± 0.49 | 3.8129 | **.001** | 0.41 | 0.19 to 0.63 |
|  | Walking | 3.96 ± 0.47 | 4.26 ± 0.45 | 3.1024 | **.005** | 0.30 | 0.10 to 0.51 |
|  | Control | 3.96 ± 0.47 | 4.00 ± 0.74 | 0.2529 | .803 | 0.04 | -0.31 to 0.40 |

| **Variable** | **Group** | **Pre-**  **intervention**  **（Mean±SD）** | **Post-**  **intervention**  **（Mean±SD）** | **t-value** | **p-value** | **Change** | | **Significance*** |
| --- | --- | --- | --- | --- | --- | --- | --- | --- |
|  |  |  |  |  |  | **Marginal means** | **95% confidence interval** |  |
| **WHOQOL Q1 Physical Health (scores)** | CTC12 | 26.91 ± 1.97 | 27.50 ± 1.74 | 2.8897 | **0.0088**** | 0.59 | 0.17 to 1.02 | ****** |
|  | TC24 | 26.96 ± 1.85 | 27.39 ± 1.56 | 2.4721 | **0.0216*** | 0.43 | 0.07 to 0.80 | ***** |
|  | SD | 26.77 ± 3.05 | 28.00 ± 2.49 | 3.3539 | **0.0030**** | 1.23 | 0.48 to 1.99 | ****** |
|  | Walking | 26.96 ± 2.69 | 27.39 ± 2.39 | 2.5543 | **0.0181*** | 0.43 | 0.09 to 0.87 | ***** |
|  | Control | 27.00 ± 2.07 | 26.13 ± 2.32 | -2.3615 | **0.0275*** | -0.81 | -1.63 to -0.11 | ***** |
| **WHOQOL Q2 Psychological Health (scores)** | CTC12 | 24.78 ± 2.02 | 26.14 ± 1.98 | 4.2712 | **0.0003***** | 1.36 | 0.70 to 2.03 | ******* |
|  | TC24 | 24.82 ± 1.61 | 25.78 ± 1.44 | 2.5911 | **0.0167*** | 0.96 | 0.19 to 1.72 | ***** |
|  | SD | 24.81 ± 1.79 | 25.91 ± 1.44 | 4.2941 | **0.0003***** | 1.10 | 0.56 to 1.62 | ******* |
|  | Walking | 24.78 ± 2.19 | 25.35 ± 1.80 | 2.8680 | **0.0089**** | 0.57 | 0.16 to 0.97 | ****** |
|  | Control | 24.83 ± 2.01 | 24.44 ± 1.67 | -1.8166 | 0.0829 | -0.39 | -0.84 to 0.06 | ns |
| **WHOQOL Q3 Social Relationships (scores)** | CTC12 | 11.64 ± 1.33 | 11.77 ± 1.41 | 0.5684 | 0.5758 | 0.13 | -0.36 to 0.64 | ns |
|  | TC24 | 11.61 ± 1.37 | 11.65 ± 1.23 | 0.2246 | 0.8243 | 0.04 | -0.36 to 0.44 | ns |
|  | SD | 11.64 ± 1.18 | 12.27 ± 1.03 | 3.3086 | **0.0033**** | 0.63 | 0.24 to 1.04 | ****** |
|  | Walking | 11.65 ± 1.34 | 11.83 ± 1.19 | 1.0000 | 0.3282 | 0.18 | -0.19 to 0.53 | ns |
|  | Control | 11.61 ± 1.50 | 11.30 ± 1.22 | -1.9084 | 0.0695 | -0.31 | -0.64 to 0.03 | ns |
| **WHOQOL Q4 Environment (scores)** | CTC12 | 31.55 ± 3.89 | 31.45 ± 3.31 | -0.3265 | 0.7473 | -0.10 | -0.67 to 0.49 | ns |
|  | TC24 | 31.35 ± 3.60 | 31.65 ± 2.60 | 0.4375 | 0.6660 | 0.30 | -1.14 to 1.75 | ns |
|  | SD | 31.32 ± 3.46 | 30.55 ± 3.86 | -2.0784 | 0.0501 | -0.77 | -1.55 to 0.00 | ns |
|  | Walking | 30.96 ± 3.86 | 30.78 ± 3.72 | -0.5462 | 0.5904 | -0.18 | -0.83 to 0.49 | ns |
|  | Control | 31.09 ± 3.00 | 31.48 ± 3.22 | 1.8993 | 0.0707 | 0.39 | -0.04 to 0.82 | ns |
| **WHOQOL Q5 Self-Perceived Health (scores)** | CTC12 | 3.55 ± 0.51 | 3.95 ± 0.58 | 3.8129 | **0.0010**** | 0.40 | 0.19 to 0.63 | ****** |
|  | TC24 | 3.57 ± 0.51 | 3.87 ± 0.55 | 3.1024 | **0.0052**** | 0.30 | 0.10 to 0.51 | ****** |
|  | SD | 3.64 ± 0.49 | 4.00 ± 0.44 | 3.4641 | **0.0023**** | 0.36 | 0.15 to 0.58 | ****** |
|  | Walking | 3.65 ± 0.49 | 3.91 ± 0.67 | 2.7865 | **0.0108*** | 0.26 | 0.07 to 0.46 | ***** |
|  | Control | 3.65 ± 0.49 | 3.57 ± 0.59 | -0.4917 | 0.6278 | -0.08 | -0.45 to 0.28 | ns |
| **WHOQOL Q6 Self-rated Overall Quality of Life (scores)** | CTC12 | 3.95 ± 0.58 | 4.41 ± 0.59 | 3.5784 | **0.0018**** | 0.46 | 0.19 to 0.72 | ****** |
|  | TC24 | 3.96 ± 0.56 | 4.35 ± 0.57 | 3.2188 | **0.0040**** | 0.39 | 0.14 to 0.64 | ****** |
|  | SD | 3.95 ± 0.58 | 4.36 ± 0.49 | 3.8129 | **0.0010**** | 0.41 | 0.19 to 0.63 | ****** |
|  | Walking | 3.96 ± 0.47 | 4.26 ± 0.45 | 3.1024 | **0.0052**** | 0.30 | 0.10 to 0.51 | ****** |
|  | Control | 3.96 ± 0.47 | 4.00 ± 0.74 | 0.2529 | 0.8027 | 0.04 | -0.31 to 0.40 | ns |

**Note:** Values are presented as mean ± SD. Changes reflect post-intervention minus pre-intervention values. 95% confidence intervals (CI) are shown for marginal means. WHOQOL Q1 = Physical Health; Q2 = Psychological Health; Q3 = Social Relationships; Q4 = Environment; Q5 = Self-Perceived Health; Q6 = Self-rated Overall Quality of Life. Significance levels: ns = not significant; *P* < .05; *P* < .01; *P* < .001. Group abbreviations as previously defined.

**Table S2. Between-group comparisons of WHOQOL subdomains after intervention.**

| **Variable** | **CTC12**  **(n = 22)** | **TC24**  **(n = 23)** | **Square Dance**  **(n = 22)** | **Walking**  **(n = 23)** | **Control**  **(n = 23)** | **F-value** | **p-value** | **η²** | **Post-hoc** |
| --- | --- | --- | --- | --- | --- | --- | --- | --- | --- |
| **WHOQOL Q1 Physical Health (scores)** | 27.50 ± 1.74 | 27.39 ± 1.56 | 28.00 ± 2.49 | 27.35 ± 2.44 | 26.13 ± 2.32 | 2.35 | .059 | 0.08 | Square Dance > Control |
| **WHOQOL Q2 Psychological Health (scores)** | 26.14 ± 1.98 | 25.78 ± 1.44 | 26.00 ± 1.23 | 25.35 ± 1.80 | 24.43 ± 1.67 | 3.94 | **.005** | 0.127 | CTC12, Square Dance > Control |
| **WHOQOL Q3 Social Relationships (scores)** | 11.77 ± 1.41 | 11.65 ± 1.23 | 12.27 ± 1.03 | 11.83 ± 1.19 | 11.30 ± 1.22 | 1.83 | .129 | 0.063 | ns |
| **WHOQOL Q4 Environment (scores)** | 31.45 ± 3.31 | 31.65 ± 2.60 | 30.55 ± 3.86 | 30.78 ± 3.72 | 31.39 ± 3.31 | 0.44 | .778 | 0.016 | ns |
| **WHOQOL Self-Perceived Health (scores)** | 3.95 ± 0.58 | 3.87 ± 0.55 | 4.00 ± 0.44 | 3.91 ± 0.67 | 3.57 ± 0.59 | 2.08 | .089 | 0.071 | n.s. (trend) |
| **WHOQOL Self-rated Overall Quality of Life (scores)** | 4.41 ± 0.59 | 4.35 ± 0.57 | 4.36 ± 0.49 | 4.26 ± 0.45 | 4.00 ± 0.74 | 1.82 | .130 | 0.0632 | n.s. (trend) |

**Note :**Values are presented as mean ± Square Dance. F-values and p-values were obtained from one-way ANOVA. η² indicates partial eta squared effect sizes. Post-hoc comparisons were conducted using Tukey’s test. WHOQOL subdomains: Q1 = Physical Health; Q2 = Psychological Health; Q3 = Social Relationships; Q4 = Environment; Q5 = Self-Perceived Health; Q6 = Self-rated Overall Quality of Life. Significance levels: *P* < .05; *P* < .01; ns = not significant. Group abbreviations as previously defined.


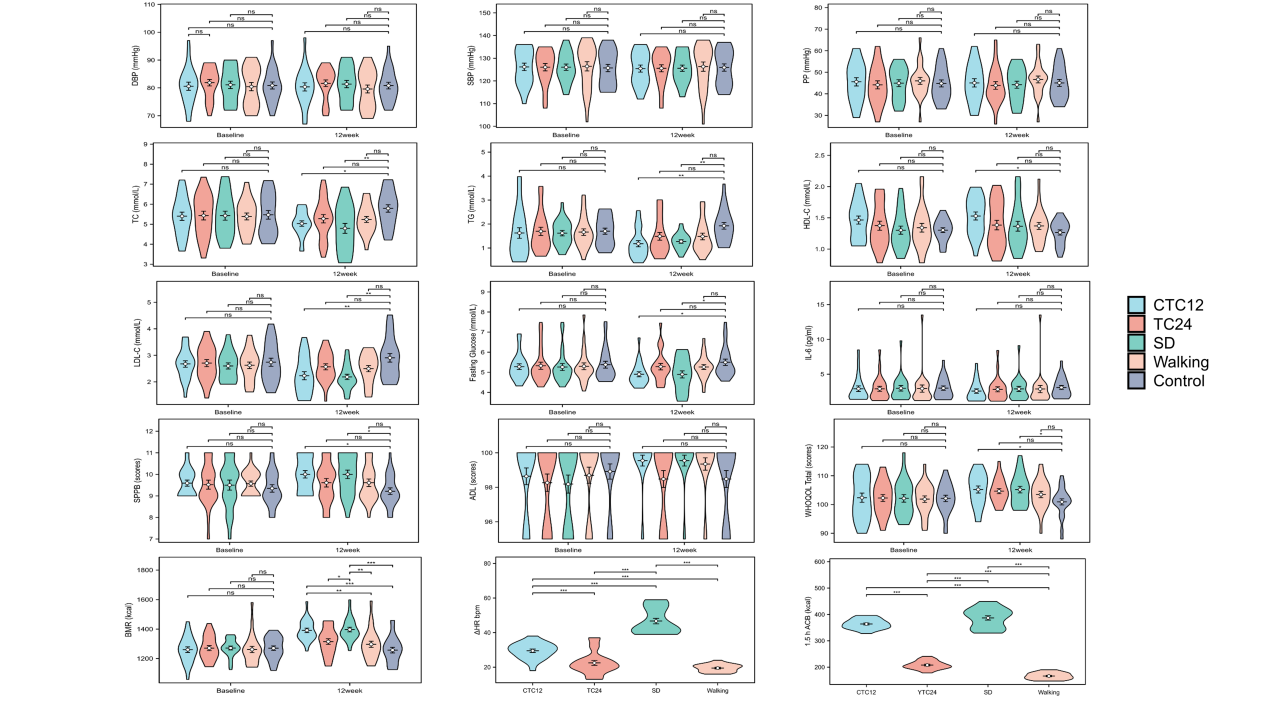


**Figure S1. Between-group comparisons of post-intervention outcomes among five intervention arms.**

**Legends:** Abbreviations:CTC12 = 12-form Chen-style Tai Chi; TC24 = 24-form simplified Tai Chi; BMI = body mass index; SBP = systolic blood pressure; DBP = diastolic blood pressure; PP = pulse pressure; TC = total cholesterol; TG = triglycerides; HDL-C = high-density lipoprotein cholesterol; LDL-C = low-density lipoprotein cholesterol; FPG = fasting plasma glucose; IL-6 = interleukin-6; BMR = basal metabolic rate; ΔHR = heart rate increase = average exercise heart rate minus resting heart rate; Calories at Rest = estimated resting energy expenditure; 1.5-hour Additional Calories Burned = additional calories expended during 90-minute exercise sessions; SPPB = Short Physical Performance Battery; ADL = activities of daily living; WHOQOL = World Health Organization Quality of Life questionnaire; bpm = beats per minute; kcal = kilocalorie.


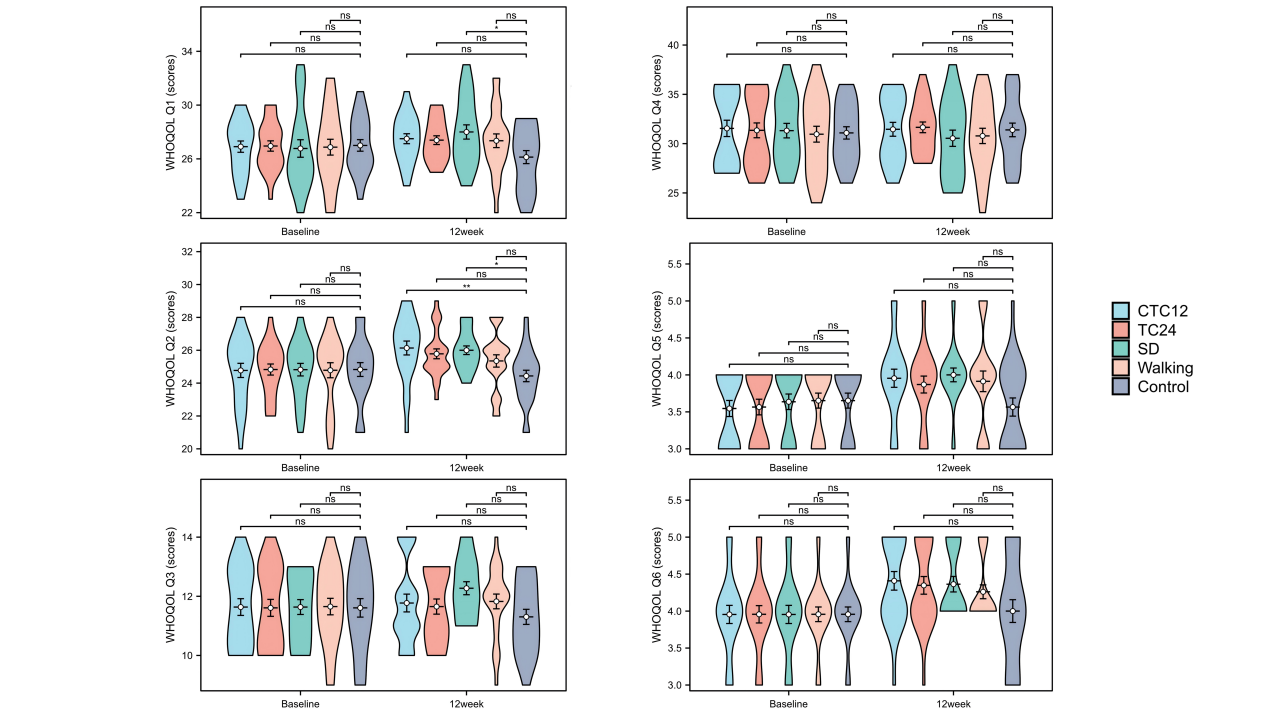


**Figure S2. Between-group comparisons of WHOQOL subdomains after intervention**

**Legends:** WHOQOL subdomains: Q1 = Physical Health; Q2 = Psychological Health; Q3 = Social Relationships; Q4 = Environment; Q5 = Self-Perceived Health; Q6 = Self-rated Overall Quality of Life. Significance levels: *P* < .05; *P* < .01; ns = not significant. Group abbreviations as previously defined.
